# Time-correlated single molecule localization microscopy enhances resolution and fidelity

**DOI:** 10.1038/s41598-020-72812-y

**Published:** 2020-10-01

**Authors:** Kobi Hermon, Shachar Schidorsky, Yair Razvag, Oren Yakovian, Eilon Sherman

**Affiliations:** Racah Institute of Physics, The Hebrew University, 91904 Jerusalem, Israel

**Keywords:** Biological sciences, Biophysics

## Abstract

Single-molecule-localization-microscopy (SMLM) enables superresolution imaging of biological samples down to ~ 10–20 nm and in single molecule detail. However, common SMLM reconstruction largely disregards information embedded in the entire intensity trajectories of individual emitters. Here, we develop and demonstrate an approach, termed time-correlated-SMLM (tcSMLM), that uses such information for enhancing SMLM reconstruction. Specifically, tcSMLM is shown to increase the spatial resolution and fidelity of SMLM reconstruction of both simulated and experimental data; esp. upon acquisition under stringent conditions of low SNR, high acquisition rate and high density of emitters. We further provide detailed guidelines and optimization procedures for effectively applying tcSMLM to data of choice. Importantly, our approach can be readily added in tandem to multiple SMLM and related superresolution reconstruction algorithms. Thus, we expect that our approach will become an effective and readily accessible tool for enhancing SMLM and superresolution imaging.

## Introduction

Recent breakthroughs in optical microscopy have allowed to visualize biological samples below the diffraction limit of light. One such approach is known as single molecule localization microscopy (SMLM) and includes PALM^1^, FPALM^2^, STORM^3^, dSTORM^4^ and their variants. SMLM uses the photoactivation or photoswitching properties of fluorophores^5–8^. Regularly, these fluorophores are used to specifically label proteins of interest within a cell either as chimeric proteins with photoactivatable fluorescent proteins (PAFPs; in PALM) or as synthetic labels of primary or secondary antibodies (STORM). The photoactivation properties of these fluorophores are used to lower the effective density of visible fluorophores such that their detected intensity profile (i.e. their point-spread function; PSF) can be well separated over space and time. The time-dependent intensity trajectories of the individual fluorophores are captured in wide-field using an imaging detector such as EMCCD or sCMOS. Then, an SMLM reconstruction algorithm is used to localize individual emitters beyond the diffraction limit, and up to a resolution of ~ 10–20nm^9^.

By now, a large number of SMLM algorithms have been demonstrated^10,11^. Traditionally, the single molecule reconstruction algorithms fit Gaussians to individual intensity peaks that are related to sparsely detected molecules in each frame of the acquired movie. These detected peaks can then be grouped over space and time to estimate the location of the individual emitters. Last, all of the emitter localizations are typically collapsed onto a single super-resolved image, having single molecular detail. The resolution of this image is defined by the localization precision, depending on the photon budget (signal and background) and system noises^9^.

All fluorophores differ in their photophysical properties, esp. in their intensity fluctuations, decay and lifetime. Thus, such properties may be indicative of the presence of individual fluorophores. Still, most current SMLM techniques are performed on individually detected peaks in individual frames. Thus, they do not consider information embedded in the time-dependent intensity of the emitters. In contrast to these techniques, methods such as Super-resolution Optical Fluctuation Imaging (SOFI)^12^ use temporal intensity statistics, namely high-order moments, to provide super-resolved images. A few recent methods use statistical analysis of intensity fluctuations, among them MUSICAL^13^, SPARCOM^14^, 3B^15^, DeconSTORM^16^, BaLM^17^ and HAWK^18^. Important advantages of temporal information-based super resolutions methods include their improved SNR, relative simplicity and potentially faster acquisition. Though some methods do show a resolution enhancement approaching to SMLM, most are limited to a resolution of ~ 50 nm. More importantly, most temporal statistics based methods provide a super-resolved image (regularly based on deconvolution), yet do not provide the explicit localizations of individual emitters. So far, a few SMLM algorithms have attempted at using temporal information toward single molecule localization. Some use SOFI and SMLM separately: one to lower the effects of over-counting and under-counting issues in SMLM^19^; and the other to enhance signal to noise (SNR) in SMLM^20^, while HAWK^17^ is used to decrease the density in the dataset before fitting. Others showed, only in principle, the efficiency of using temporal statistics to enhance identification of clustering artifacts^21^, acquisition time^22^, and counting precision^8^.

Here, we use the temporal information that is associated with the individual fluorophores detected via SMLM to enhance its reconstruction performance. Specifically, we consider the temporal decay in the intensity of the fluorophores and perform SMLM on modified PSFs that contain this information. We term the new dataset 'time-correlated-data' (tcData). As in other temporal-based datasets, imaging conditions suitable for SOFI reconstruction^12^ are sufficient for our approach. Thus, we perform SMLM reconstruction over tcData, and term our approach 'time-correlated-SMLM' (tcSMLM). The advantages of using statistics of temporal fluctuations and the high spatial resolution provided by SMLM are inherent to tcSMLM. We use extensive simulations and experiments to show that our approach results in improved resolution and higher fidelity of reconstructed images. Our tested cases include multiple samples, fluorophore types and publically available datasets.

The enhancement in reconstruction performance of our approach was most prominent under conditions of high fluorophore density, poor signal to background (e.g. from out-of-focus emission), and high frame-rate imaging that are typically problematic for traditional SMLM reconstruction approaches. Moreover, our approach can be utilized in tandem with additional (and likely, any) SMLM reconstruction algorithms that do not already utilize 'deep' (i.e. more than 2 frames) temporal fluctuations of the emitters. For instance, such algorithms may utilize deconvolution techniques (e.g. DeconSTORM becoming 'tcDeconSTORM' when applied to tcData) or spatial correlations (e.g. SRRF^23^, likewise becoming 'tcSRRF'). Thus, we provide a versatile and user-friendly add-on tool for the enhancement of SMLM reconstruction, esp. under stringent imaging conditions.

## Results

### The algorithm of time correlated SMLM (tcSMLM)

**Figure 1:**
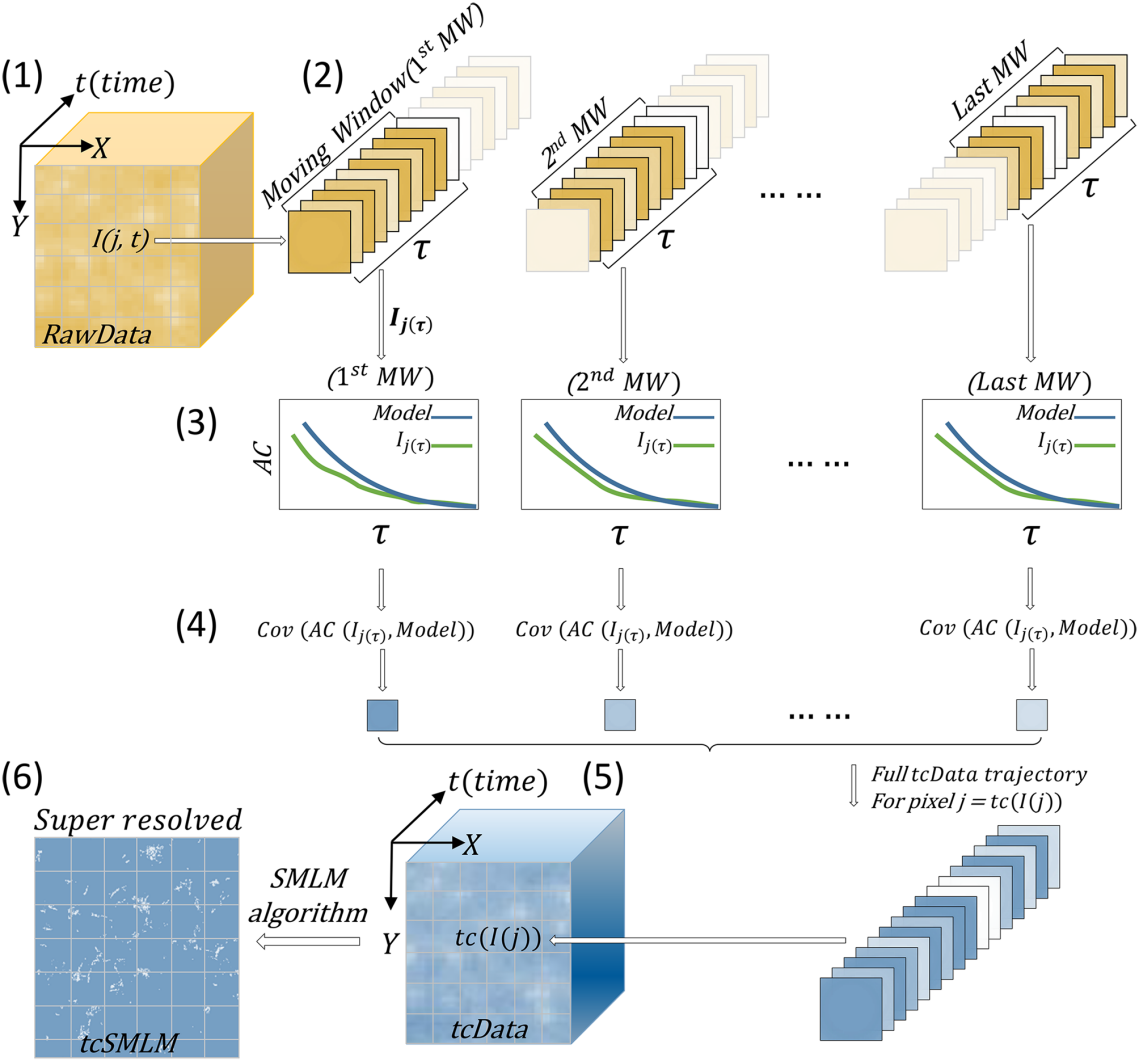
A schematic description of time-correlated single molecule localization microscopy (tcSMLM). A scheme that describes time-correlated single molecule localization microscopy (tcSMLM) is provided. See main text for a detailed description of the algorithmic steps.

We provide below a schematic description of our approach for decoding SMLM using time correlations, which we refer to as 'time correlated SMLM' (tcSMLM). Intuitively, each PSF that belongs to a single fluorophore has a specific temporal dependence. This dependence can be captured as decay in the temporal correlation of its intensity. Considering that, we compare the correlation decay for each PSF in the acquired data to a common model—the characteristic correlation decay of the fluorophore under study. Thus, temporal information is added to the spatial data during the reconstruction process. We then show that such reconstruction can improve the localization performance of the individual PSFs. We next describe a detailed scheme of our algorithm in Fig. 1, and through the following steps:

1. We start with acquiring the fluorescence intensity of single photoactivatable fluorescent emitters through SMLM imaging. We refer to this time-dependent intensity data as ‘RawData’, for simplicity (Fig. 1, item (1)). Then, we choose a 'Moving Window' (MW) with size τ (Fig. 1(2)), which is associated with a specific imaged fluorophore. More details, considerations and analyses regarding the MW choice are provided later in the main and supplemental text (see Fig. 6 and Figs. S1, S2). Next, we consider a complete time trajectory in the RawData, I(j,t) (Fig. 1(1)), of length N, where N is the number of frames in RawData and where j is the index of a pixel of interest. We then create a time-dependent function f(t), derived either from a model of the time-dependent intensity of a typical fluorophore (see SI, note 5 and Fig. S7) or from representative experimental data.
2. We acquire a vector ${\hbox{I}}_{{\rm j}(\uptau )}$ with size τ (Fig. 1(2)), constructed from the first τ time-dependent terms in $\hbox{I}\left(\hbox{j},\hbox{t}\right)$. Similarly, we construct a vector $\hbox{f}(\uptau )$ from the first τ time-dependent terms in $\hbox{f}(\hbox{t})$.
3. Then, we perform auto-correlation ($\hbox{AC}$) of the time-dependent trajectory ${\hbox{I}}_{{\rm j}(\uptau )}$. Similarly, we perform AC of $\hbox{f}\left(\uptau \right)$ (see Fig. 1(3)). As fluorophore intensity fluctuations strongly depend on imaging conditions^7^, we used an averaged model for AC(f(t)) throughout our research. See SI, note 3.3 for a thorough description of the choice of an averaged model.
4. We calculate the covariance ($\hbox{Cov}$) of the resultant $\hbox{AC}$ signal of ${\hbox{I}}_{{\rm j}(\uptau )}$ and of $\hbox{f}(\uptau )$, namely $\hbox{Cov}[\hbox{AC}\left({\hbox{I}}_{{\rm j}\left(\uptau \right)},\hbox{ f}\left(\uptau \right)\right)]$ (see Fig. 1(4)). The Cov operation naturally selects AC functions that have a similar decay to the model, while efficiently rejecting AC functions that are very different (e.g. having a very fast or a very slow decay time; see SI, note 3 for further details). Performing the same process over the time trajectories of length τ, starting with $\hbox{t}=1$, for each pixel $\hbox{j},$ the algorithm yields a single image, corresponding to the RawData over time of 1 to τ. This image is used as a single frame in a newly reconstructed movie, as explained below.
5. Similarly, the algorithm is used to extract frames for other time points, as follows: we shift the MW sequentially one time step forward at a time, from 2 to τ+2 (i.e. time step here equals 1 frame, but note that each time step may larger). For each time step, we perform steps (3)–(4) again. Finally, after we obtain the last MW, we conclude a data structure which we refer to as the 'time-correlated Data' ($\hbox{tcData}$; Fig. 1(5)). Thus, provided we shift MW one frame ahead, the length of $\hbox{tcData}$ is N-τ. Notably, the movie embedded in $\hbox{tcData}$ now encodes similarities between the AC of the fluorophores to the model, rather than in their raw intensities.
6. The final step of reconstruction in our scheme employs detection of individual emitters in tcData, using an SMLM reconstruction algorithm of choice (Fig. 1(6)). Importantly, the SMLM algorithm can be any SMLM or super-resolution algorithm which does not rely already on time correlations for reconstruction, and can be chosen according to the user's preferences. In our analyses, we mainly employed ThunderSTORM^24^. However, we verified the wide applicability of our approach to multiple SMLM reconstruction algorithms, and further demonstrate this in our study (in Fig. 7).

### Examples of tcSMLM reconstruction of simulated and experimental data

**Figure 2:**
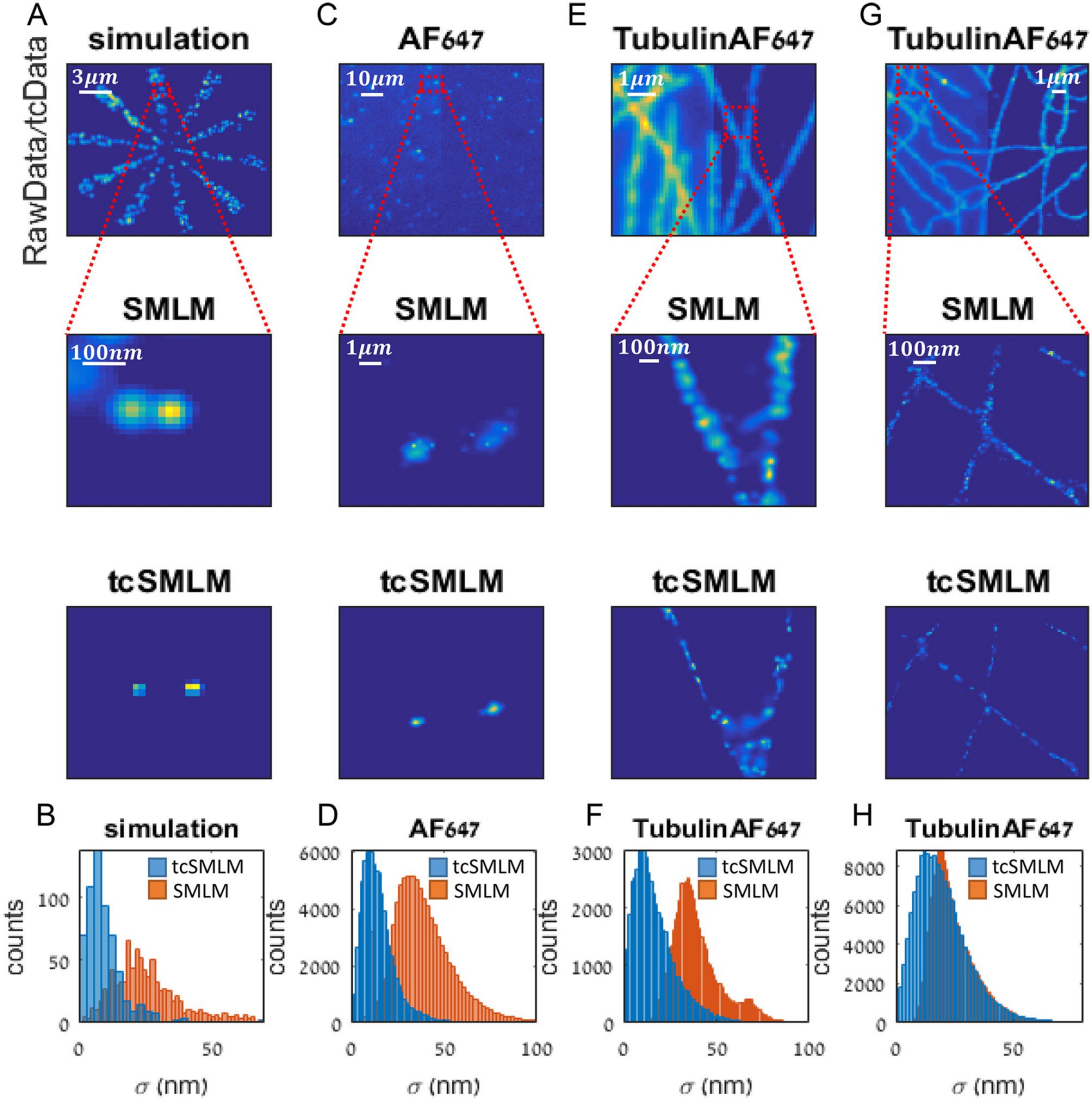
Examples of tcSMLM reconstruction of simulated and experimental data. (**A**) Simulated data of fluorophores embedded in a Siemens star (top), and zoom images after reconstruction with either SMLM (middle) or tcSMLM (bottom). (**B**) Localization errors (σ) for SMLM and tcSMLM reconstruction of the data in A. (**C**) dSTORM imaging of Alexa Fluor647 labelled antibodies scattered on a coverslip coated with PLL (top), and zoom images after reconstruction with either SMLM (middle) or tcSMLM (bottom). (**D**) Localization errors (σ) for SMLM and tcSMLM reconstruction of the data in C. (**E**) Published data dSTORM imaging of tubulin immunostained with Alexa Fluor647 labelled antibodies scattered on a coverslip coated with PLL (top), and zoom images after reconstruction with either SMLM (middle) or tcSMLM (bottom). (**F**) Localization errors (σ) for SMLM and tcSMLM reconstruction of the data in (**E**). (**G**) Published data of dSTORM imaging of Alexa Fluor 647 labelled antibodies scattered on a coverslip coated with PLL (top), and zoom images after reconstruction with either SMLM (middle) or tcSMLM (bottom). (**H**) Localization errors (σ) for SMLM and tcSMLM reconstruction of the data in (**G**).

We first demonstrate the utility of our method using simulations and experimental data (Fig. 2). Specifically, we show the narrowing of the PSF of individual fluorophores as reconstructed by tcData, in comparison to RawData. We present a comparison of distinctive details in the super-resolved images (Fig. 2A,C,E,G) and demonstrate a significant reduction in the localization uncertainty of the detected molecules (Fig. 2B,D,F,H) when using tcSMLM. We start with simulated data (Fig. 2A,B). For that, we generated simulated movie of single emitters using a published SOFI simulation tool^25^. Using the simulation, we generated 600 emitters, placed randomly within a Siemens star with 10 wings, on a 100 × 100px matrix. The parameters we chose for the simulation are based mainly on our mathematical analyses (see SI, note 3), and were as follows: frame rate of 1000 fps; density of $\sim 5\frac{\hbox{active fluorophores}}{\upmu {\hbox{m}}^{2}}$ , bleaching time 5 s, acquisition time 2 s and negligible background (see also Methods). TcSMLM shows a better resemblance to the ground truth (GT) compared to SMLM (Fig. 2A). Moreover, the uncertainty median is 9 nm for tcSMLM, compared to 22 nm for SMLM (Fig. 2C).

Next, we deposited antibodies labelled with Alexa Fluor 647 (Alexa647) on a coverslip, and imaged the sample via dSTORM imaging in TIRF mode (see Methods). We chose Alexa647 due to its long photobleaching time and its high signal-to-background ratio (SBR)^26^. The imaging was done with a standard frame rate ($20\hbox{ fps}$) to demonstrate the performance of tcSMLM under regular conditions. The sum intensity (SumImg) of tcData shows molecular aggregates distinctly separated from the background, as compared to the sum intensity of RawData (Fig. 2C). The presented zoom images of two aggregates show significant narrowing of the PSF (Fig. 2C). The uncertainty median for tcSMLM is $13\hbox{ nm}$, compared to 36 nm for SMLM (Fig. 2D).

Finally, we show in Fig. 2E–H two open-source and published examples of Tubulin molecules in microtubule filaments, stained with Alexa647^10,27^. For simplicity, we used only the initial 3000 frames of the movies. We show that tcData summing distinguishes the microtubules from the background, as compared to RawData summing (Fig. 2E,G). In the presented zoom images, the results for tcSMLM reconstruction confirm the narrowing of the PSF in comparison to SMLM. Also, in the first example (Fig. 2G), the median of the localization uncertainty for tcSMLM is 14 nm, compared to 37 nm for SMLM (Fig. 2F). For the second example of microtubule with a relatively lower density of emitters (Fig. 2G,H), we obtain a localization uncertainty median of 18 nm for tcSMLM, compared to 23 nm for SMLM. We further analysed a wide range of simulations and experimental data, and found that results vary based on frame rate, SBR and density of emitters. Below, we quantify the implications of these parameters on tcSMLM reconstruction.

### Quantitative comparison of tcSMLM and SMLM performance

**Figure 3:**
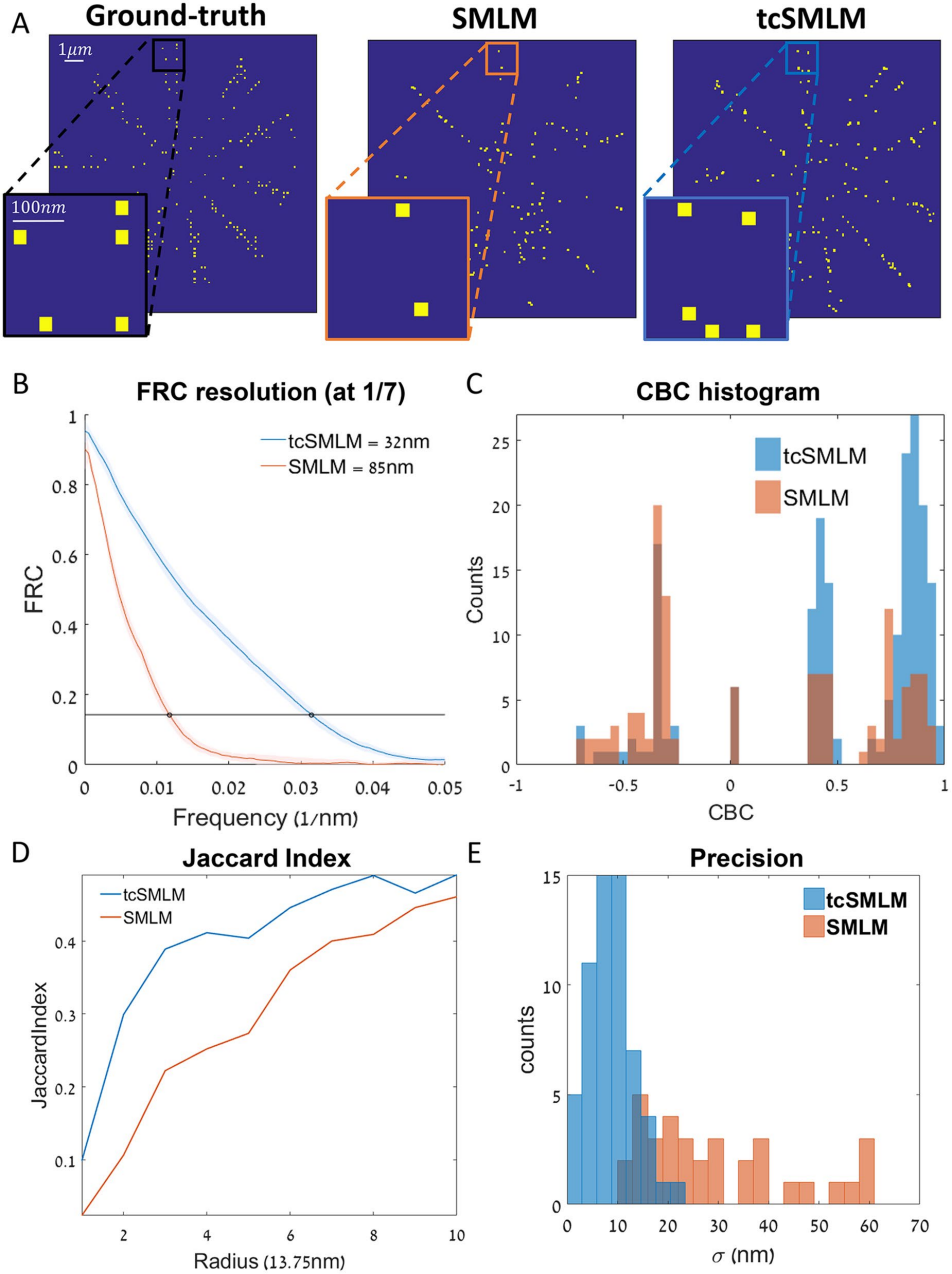
Quantitative comparison of tcSMLM and SMLM performance. (**A**) Images of simulated emitters, showing tcSMLM (right), SMLM (middle) and ground-truth (GT; left). see Methods for further details on simulation. (**B**) Fourier-ring correlations (FRC) of data in panel (**A**), comparing either SMLM and GT (red) or tcSMLM and GT (blue). FRC resolution is determined at the frequency where FRC = 1/7. (**C**) Coordinate-based co-localization (CBC) of data in panel (**A**), comparing either SMLM and GT (red) or tcSMLM and GT (blue). (**D**) Jaccard Index of data in panel A, comparing either tcSMLM and GT (blue) or SMLM and GT (red), vs. 10 radii values (13.75 nm to 137.5 nm). (**E**) Precision histogram of data in panel (**A**), where blue columns represent TP counts of tcSMLM vs. their localization error and red columns represent TP counts of SMLM vs. their localization error.

In order to quantify the reconstruction performance of tcSMLM relative to SMLM, we compared the resolution and fidelity of these approaches when applied to simulated data (Fig. 3). Specifically, we chose published analyses: Fourier Ring Correlation (FRC;^9,28^), Coordinate-Based Colocalization (CBC;^29^), Jaccard Index and a measure of precision^11^.

Starting with the comparison of resolution, we generally expect a resolution enhancement of tcSMLM relative to SMLM. The reason is that tcSMLM employs autocorrelation of intensity data. This autocorrelation is also used by 2nd order SOFI reconstruction, which effectively shrinks the PSF by a factor of $\surd 2$ relative to the intensity image (i.e. our RawData;^30^]. To evaluate the resolution enhancement by tcSMLM over SMLM reconstruction, we simulated multiple individual fluorophores on a field of view using a published simulation tool^25^. Our simulated conditions were chosen to demonstrate the pros of tcSMLM (See SI, Note 3.1), and included 250 emitters over a Siemens star with 10 narrow wings, 25 × 25 pixels (effective 165 nm per pixel), fast acquisition rate of 2000 fps, bleaching time of 5 s, acquisition time of 2 s, on state 180 ms and off state 120 ms. Thus, our simulation emulate a high density sample where for each diffraction limited area, there are ~ 5–50 active fluorophores (Here, ~ 5).

In our simulations we further modified multiple parameters, including fluorophore density, bleaching and blinking statistics, background conditions, imaging frame-rate and peak intensity. We evaluated the relative performance of tcSMLM under these varying conditions using Fourier ring correlation statistics (FRC) and precision. Typically, FRC provides a measure of the resolution of an image when compared to the ground-truth data. The resolution is considered as the inverse of the cutoff frequency at which the FRC curve drops to a value of 1/7^9^. In the presented simulation (Fig. 3A), the FRC curve of dSTORM (Fig. 3B; orange curve) showed a faster drop in comparison to the FRC curve of tcSMLM (Fig. 3B; blue curve). Using the accepted FRC cutoff criterion, the FRC resolution of tcSMLM was 32 nm, in comparison to 85 nm for SMLM. Thus, tcSMLM outperforms SMLM reconstruction via ThunderSTORM in terms of spatial (FRC) resolution. Generally, the conditions for SMLM reconstruction result in some blurring of the emitters (as in Fig. 2A,C,E,G), as compared to tcSMLM, which showed more isolated emitters within the simulated patterns.

In order to quantify the fidelity of our approach we used coordinate based colocalization (CBC), Jaccard Index and precision. Coordinate based colocalization has been developed to quantitatively evaluate the fidelity of a reconstruction approach relative to the ground-truth (GT). It yields a comparative image in which the colocalization of the reconstructed image and GT is highlighted with values ranging from − 1 (anticorrelated) to 1 (completely correlated). Here, the values of the test image are shown as a histogram (Fig. 3C). The histogram of the CBC analysis for tcSMLM has significantly higher correlation for tcSMLM compared to SMLM.

Jaccard Index (JI) is a common fidelity measure: the number of true positive (TP) detections divided by the sum of TP, false negative (FN) and false positive (FP) detections [i.e., (TP + FN + FP)]. The TP, FN, FP values were evaluated for a range of radii around each emitter’s location in the GT, and compared to the same matrix in the super resolved image. As shown in Fig. 3D, JI for tcSMLM was significantly higher for all 1–10 radii values (i.e. 13.5–135 nm).

Our measure of precision is the evaluation of the median of the localization uncertainty only for the TP detections. This measure of is used to quantitatively measure the fidelity of a reconstruction approach relative to the GT. Moreover, inaccurate results tend to have higher uncertainty values. Therefore, our measure also neglects artificial suspicions. Figure 3E shows higher counts for tcSMLM relative to SMLM (i.e. $T{P}_{tcSMLM}>T{P}_{SMLM}$) as well as significantly lower uncertainty values. In conclusion, all three fidelity measures show higher fidelity values for the specific simulation shown in Fig. 3 for tcMLM reconstruction vs. SMLM reconstruction. Next, we scanned a wide range of conditions to evaluate the performance and robustness of our method.

### TcSMLM resolution enhancement under varying signal-to-background and density conditions

**Figure 4:**
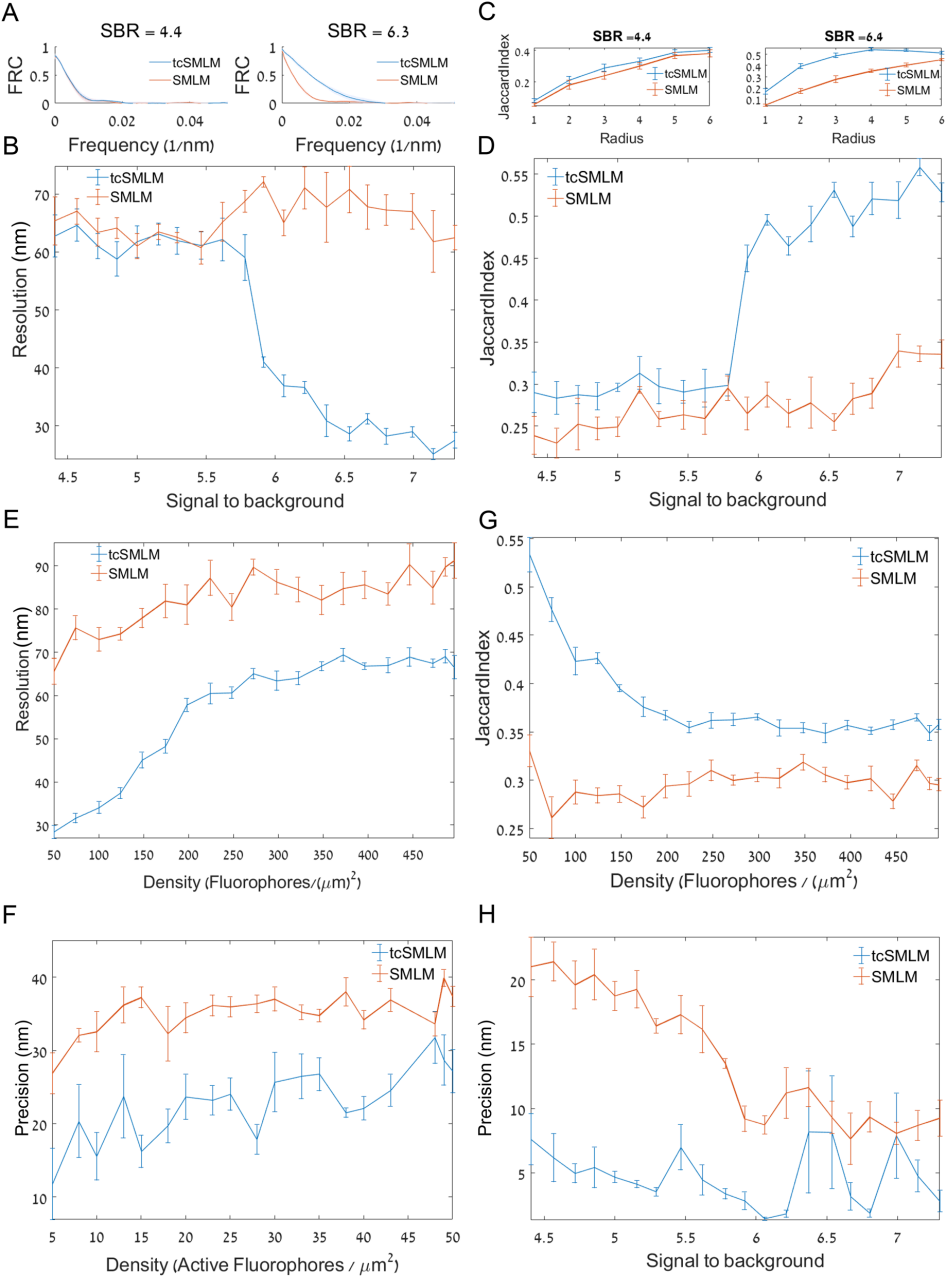
tcSMLM resolution enhancement under varying signal-to-background and density conditions. (**A**) FRC of simulated emitters at SNR levels of 4.4 (left) and 7.3 (right). Results are shown for SMLM (red) and tcSMLM (blue) reconstructions. (**B**) FRC resolution (as defined by the frequency where FRC = 1/7), as a function of SBR. (**C**) CBC of simulated emitters at SBR levels of 4.4 (left) and 7.3 (right). Results are shown for SMLM (red) and tcSMLM (blue) reconstructions. (**D**) Jaccard Index as a function of SBR. Results are shown for tcSMLM (blue) and SMLM (red). (**E**) FRC resolution (as defined by the frequency where FRC = 1/7) of simulated emitters at densities between 50 and 100 emitters/μm^2^ where ~ 5–10% are active in each frame. Results are shown for SMLM (red) and tcSMLM (blue) reconstructions. (**F**) Precision (as defined by the mean of localization errors for TP values) of simulated emitters vs. density (as in **E**). Results are shown for SMLM (red) and tcSMLM (blue) reconstructions. (**G**) Jaccard index of simulated emitters vs. density (as in **E**). Results are shown for SMLM (red) and tcSMLM (blue) reconstructions. (**H**) Precision (as defined by the mean of localization errors for TP values) of simulated emitters vs. SBR. Results are shown for SMLM (red) and tcSMLM (blue) reconstructions.

SMLM reconstruction is often sensitive to the SBR of the individual emitters. Background can originate from out-of-focus fluorescence and sample contaminations. Thus, we simulated the background by considering various levels of out-of-focus emission. Such a background is particularly hard to filter-out by methods relying on time-correlation of intensity fluctuations (see SI, note 3.1). To study the effect of SBR on tcSMLM reconstruction, we used simulated emitters, with a constant frame rate of 1000 fps and typical density of $\sim 100\frac{\hbox{emitters}}{\upmu {\hbox{m}}^{2}}$ (with ~ 5–10% active emitters per $\upmu {\hbox{m}}^{2}$) while varying the background levels, and thus, under a range of SBR conditions (Fig. 4A–D). Other temporal parameters as in Fig. 3. We noted that tcSMLM showed an improvement in (FRC) resolution at relatively high SBR values (7.3), but not at lower SBR of 4.4 (Fig. 4A,B). A detailed study showed a cutoff of resolution enhancement above SBR of ~ 5.7. Similar results were obtained using the test of reconstruction fidelity via JI. The JI test showed significant improvement by tcSMLM over SMLM reconstruction above SBR of ~ 5.7 (Fig. 4C,D), which matches our results for FRC resolution as a function of SBR (compare Fig. 4B,D).

Emitter density also affects strongly the performance of SMLM reconstruction. While initially demonstrated on very sparse images^1,31^, recently published algorithms have been devoted to improvements of SMLM performance for reconstructing multiple, partially overlapping emitters^16,23,32^. To test this effect on tcSMLM enhancement over SMLM reconstruction, we varied the density of emitters in the simulations ($50{-}500\frac{\hbox{fluorophores}}{\upmu {\hbox{m}}^{2}}$ of which ~ 5–10% active emitters per $\upmu {\hbox{m}}^{2}$) while keeping fixed parameters of SBR = 6 and frame rate of 1000 fps. Strikingly, tcSMLM outperformed SMLM reconstruction under the entire range of emitter densities, in terms of FRC resolution (Fig. 4E) and especially in terms of fidelity as determined by JI analyses for a fixed radius of $2$ (Fig. 4G) and accuracy vs. SBR and density (Fig. 4F,H). We conclude that tcSMLM shows dramatic enhancement over SMLM reconstruction under high SBR conditions, and regardless of emitter density.

### TcSMLM resolution enhancement dependence of the window size

Since our algorithm uses temporal correlations in the acquired raw data, it naturally depends on the size of our temporal moving window over which the tcSMLM is employed (see SI, notes 1, 3.1). To study this effect on tcSMLM performance (as compared to SMLM), we simulated emitters with the following parameters: a fixed density of $100\frac{\hbox{emitters}}{\upmu {\hbox{m}}^{2}}$, frame-rate of 1000 fps, low SBR of 0.03 and other temporal values as in Fig. 3. Next, we scanned the size of the temporal moving window (MW) between 5 and 250 frames and recorded the Jaccard Index and FRC resolution for each MW.

**Figure 5:**
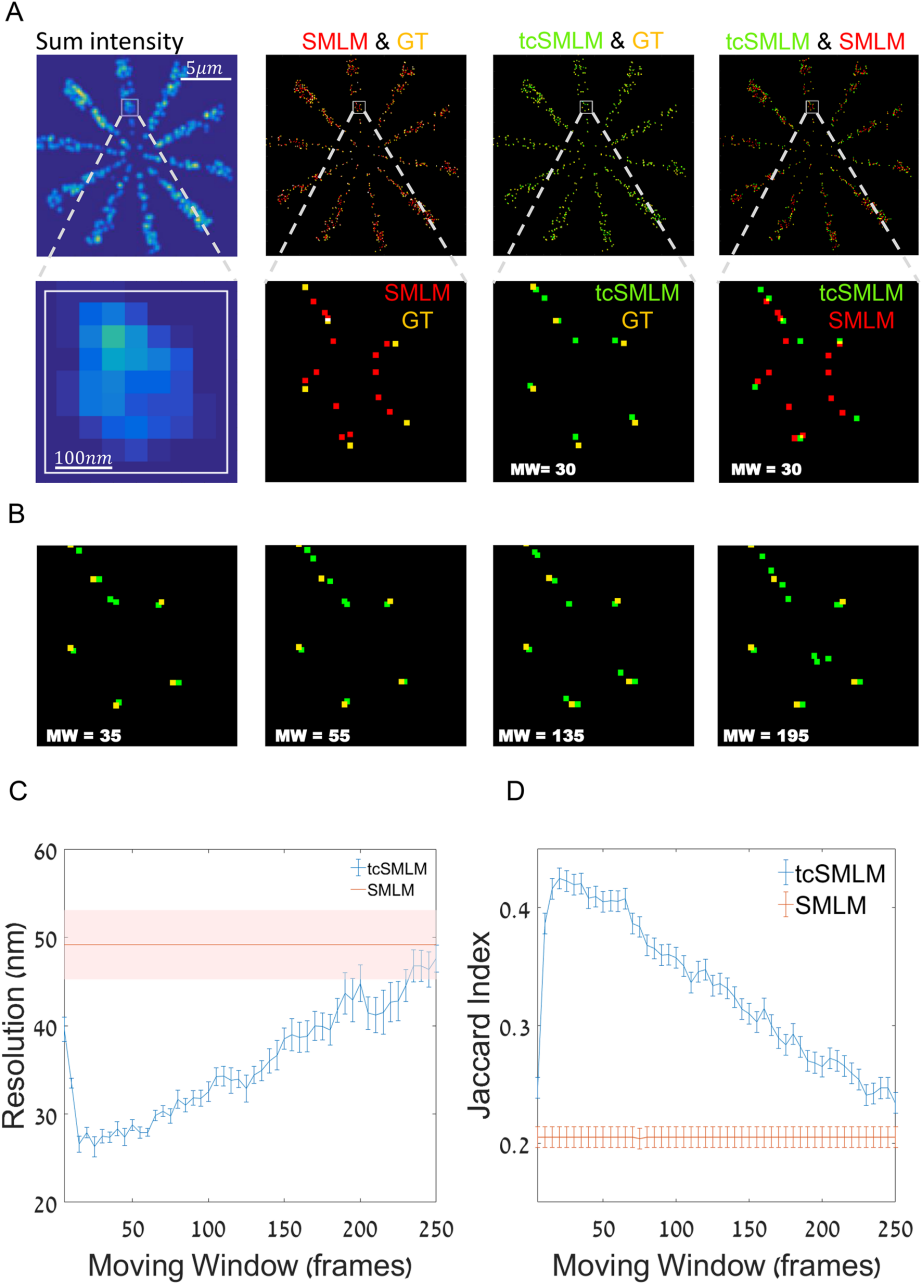
The dependence of tcSMLM resolution enhancement on reconstruction window-size. (**A**) Simulated data of fluorophores embedded in a Siemens star (left column; top), and zoom images (left column; bottom). Shown are also comparisons of ground-truth (GT) and SMLM (second column), GT and tcSMLM (third column) and tcSMLM and SMLM (right column). (**B**) A comparison of GT and tcSMLM-reconstruction of the data in (**A**) (zoom image) using moving windows of 35, 55, 135 and 195 frames. (**C**) FRC resolution dependence on the size of the moving window. FRC resolution of SMLM is independent of the window size and shown in red. (**D**) Jaccard index dependence on the size of the moving window. The Jaccard Index of SMLM is independent of the window size and shown in red.

We start by demonstrating visually the effect of the MW size. For that, we recorded the images for MW = 30, 35, 55, 135 and 195. We superimposed these images with the GT data, where the results of tcSMLM are shown in green, SMLM in red and both are shown in relation to the GT in orange (Fig. 5A,B). We found that $\hbox{MW}\approx 30$ best matched the GT in terms of number and fidelity of localizations out of the tested MWs. Last, a merged (green and red) image of SMLM and tcSMLM is provided, showing the relative performance of these reconstruction algorithms. We also show that the fidelity of detection generally decreased with the size of the temporal window, above 35 frames (Fig. 5B).

We next show the FRC resolution and JI as a function of MW (Fig. 5C,D). We found that tcSMLM performance was optimal using MW of ~ 20–30 frames. Importantly, the optimal MW matched in size the typical decay in the correlation time of the emitters. Here, we chose the following photophysical parameters for the fluorophores: on-time = $180\hbox{ ms}$, off-time = $120\hbox{ ms}$ and an acquisition rate of 1000 fps. We chose here a time step of 2 frames for tcData, and therefore the optimal MW should be, according to theory: ≈ $\frac{180\cdot 120}{2\cdot (180+120)}=36$ which is confirmed by our results.

Even though FRC resolution and reconstruction fidelity for tcSMLM (as determined by JI) showed the best performance using specific window sizes, they outperform SMLM reconstruction regardless of the window size across the MW range presented (Fig. 5C,D). These results can be explained as follows. Very short MW results in reconstruction close to standard SMLM (i.e. as reconstruction is only employed on tcData). Very long MW captures only the bleaching decay process, and therefore results either in less emitters reconstructed or in false-positive data emerging from out-of-focus noise. In the simulation presented, the bleaching process was significantly longer than the acquisition time, and therefore we found more false-positive results as we increased the MW. We conclude that given high frame rate, stable fluorophore and in conditions of low SBR, tcSMLM shows better resolution and fidelity than SMLM, regardless of the MW size. However careful choice of MW greatly improves the results.

### Optimal MW for tcSMLM

**Figure 6:**
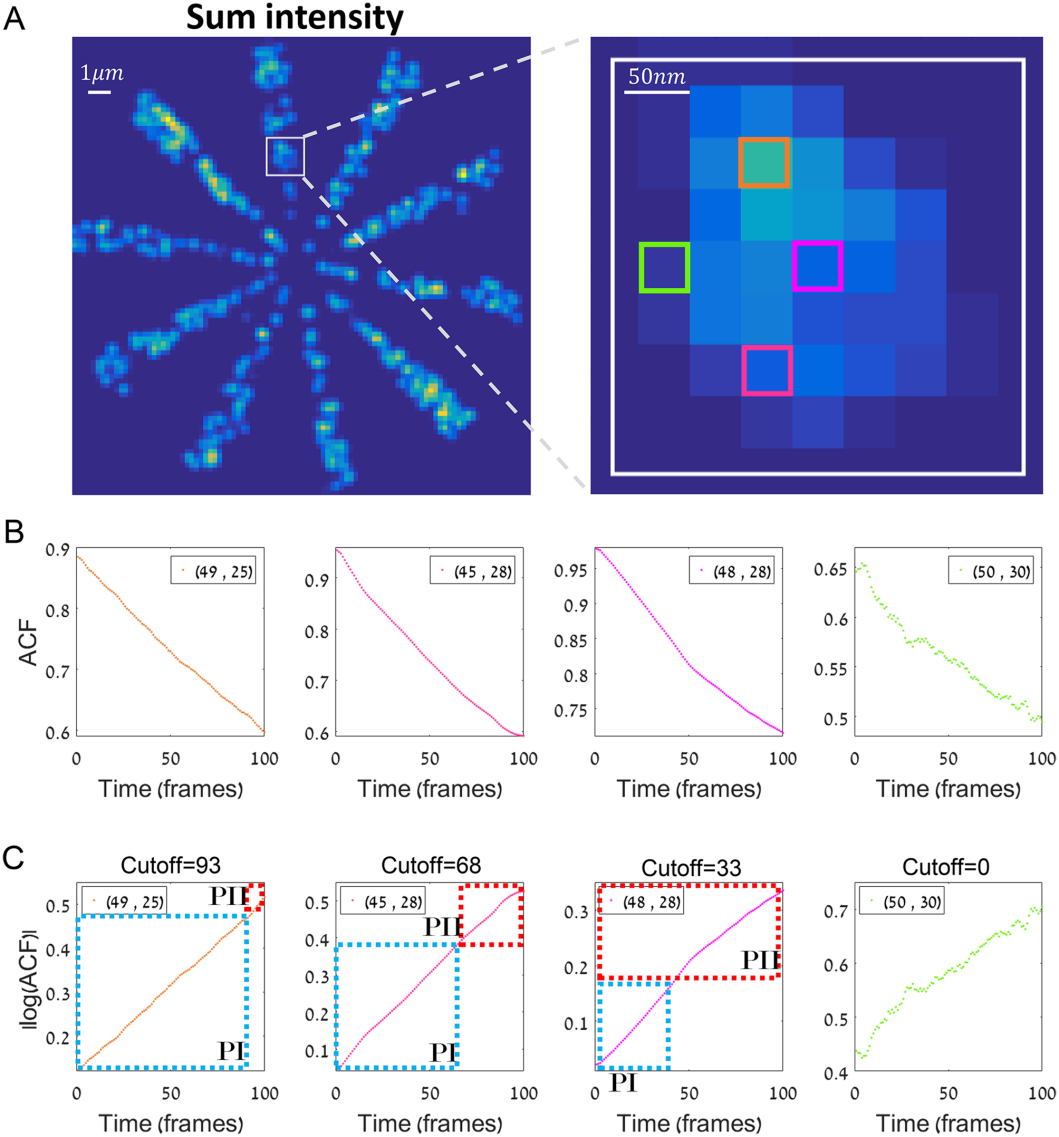
An algorithm for finding the optimal moving window. (**A**) Simulated data of fluorophores embedded in a Siemens star (left) and zoom image (right). Multiple representative pixels are highlighted in the zoom image for further analysis and comparison below. (**B**) The auto-correlation of the pixels highlighted in zoom image in (**A**). PI and PII in B,C,D, (**C**) The absolute value of the log (auto-correlation) of the pixels highlighted in zoom image in A. blue and red dotted areas, stand for the ACF decay of one emitter vs. total trajectory decay (see main text). Cutoff titles represent the ~ frame where PII starts.

Even though tcSMLM outperform SMLM over a wide range of MWs, the results may vary significantly when the chosen MW is far from optimum (e.g. Fig. 5C,D). Therefore, we wanted to establish a robust way to determine the optimal size of the MW, given a fluorophore of interest (Fig. 6). For that, we consider two decay processes that characterize the intensity trajectory of the fluorophore:

1. Process I (PI) is a decay process of fluorescence emission, either through emitters entering a prolonged dark state or photobleaching^5,8^.
2. Process II (PII) is a longer decay of the intensity, comprising out-of-focus noise and overlapping trajectories at the same diffraction spot of the fluorophore of interest, thus overlapping its signal in space and time.

These two decaying parameters are typically revealed using the individual $\hbox{ACF}$ curves of specific pixels (Fig. 6A,B;^5,33^). Considering a typical trajectory, PI depends on the frame-rate, fluorophore photophysics and imaging buffer. Since photons are emitted following a Poisson distribution, the decay of PI is exponential (Fig. 6B) with a homogenous, unique life-time based on the on- and off-times of the fluorophore of interest (Fig. 6C, graph regions surrounded by blue dotted rectangles). In contrast, PII has a wide spectrum of decay times, changing along with interferences to the intensity trajectory (Fig. 6C, regions surrounded by red dotted rectangles). Thus, we found the cut-off between PI and PII as follows:

- We chose 4 representing locations (e.g. pixels) from the sum intensity image ('sumimg') of the chosen simulation / experimental data. The higher the intensity in the 'sumimg', we expect to find more decay times following the PII process, represented as curve fluctuations in the $\hbox{ACF}$. Here, we chose 4 locations, of which one of them was expected to contain noise due to having a very low signal (Fig. 6A, green square), and the 3 others representing different levels of intensity in the 'sumimg' (Fig. 6A, orange, red and purple squares).
- We performed $\hbox{ACF}$ over the full intensity trajectory of each of the highlighted pixels. Here, we show only a minor part (first 100 frames) of the trajectory, to emphasize the difficulty in choosing the exact cut-off between PI and PII, and therefore the need for a robust method (Fig. 6C, blue vs. red surrounding rectangles of graph regions). The blue rectangles show a smooth exponential decay of the $\hbox{ACF}$, while the red rectangles show bumpier curves representing the changes in the decay times due to interferences in the trajectory. Note that the colors of the ACF curves in Fig. 6B,C below) follow the colors of the highlighted pixels in the zoom image in Fig. 6A.
- To automate the process of choosing the optimal MW size, we now focus on the $\hbox{log }(\hbox{ACF})$ curves. The smooth exponential part of the ACF curve transforms to a linear line (Fig. 6C, blue rectangles), while the rest of the curve emphasize the changes in the exponential decay (Fig. 6C, red rectangles). Moreover, we observe that the green line (Fig. 6C, green curve) significantly fluctuates from the beginning, which shows indeed that it does not arise from a process with a unique decay time throughout its trajectory; i.e. it does not represent a signal, but rather a decaying background, as we first assumed.
- Finally, in order to find the exact cut-off between PI and PII, we used the Pearson correlation to find the level of linearity in the PI regions of the log(ACF) trajectories. The cutoff was chosen at Pearson correlation $<\hspace{0.17em}$0.9. The typical times for transition from PI to PII, namely $\hbox{PI}\_\hbox{cutoff}$, were 93, 68, 33 and 0 frames for the highlighted pixels in Fig. 6A (from left to right; respectively). A $\hbox{PI}\_\hbox{cutoff}$ of 0 represents a trajectory that arises from background and does not contain a significant signal. In order to capture most emitters, we chose MW as $\hbox{MW}=\hbox{min}(\hbox{PI}\_\hbox{cutoff}(\hbox{n}))$, where n represents the number of locations chosen (e.g., 4 locations in this example). Based on this analysis for our simulated data, the appropriate MW we found was $\hbox{MW}\approx 33\hbox{ frames}$, which corroborates the results found in Fig. 5C, D. See SI note 3.1 for further details and Figs. S1–S4 that show experimental results as well.

To conclude, we provide a systematic way to find the optimal MW size per fluorophore of choice and imaging conditions. However, variability in the experimental data may require further fine-tuning and consideration of this process. Further details regarding MW optimization are described in SI note 6 and in the Methods.

### tcSMLM enhancement of super resolved DNA-origami nanoruler

So far, we have studied the validity, performance and benefit of our approach using simulations, for which ground-truth is readily available. Still, for experimental data, ground truth is rarely available. To validate our reconstruction approach, we have imaged particles of known structure, as follows. We purchased nano-rulers made of DNA origami (STORM 50R GATTAquant), labelled with Alexa647 at its ends (i.e. at a 50 nm distance between the markers)^34^. We conducted dSTORM imaging of such nano-rulers as they were tightly adhered to coverslips through biotin interactions of both of the nano-ruler ends with neutravidin-coated coverslips (see Methods). Importantly, to test the useful range of imaging conditions for tcSMLM, we conducted the imaging under a wide range of frame rates (10–252 fps). We chose ROIs of 90 × 90 pixels in our iXon^+^ Ultra EMCCD camera (Andor) with ~ 20 nano-ruler particles in each ROI (following manufacturer recommendations).

**Figure 7:**
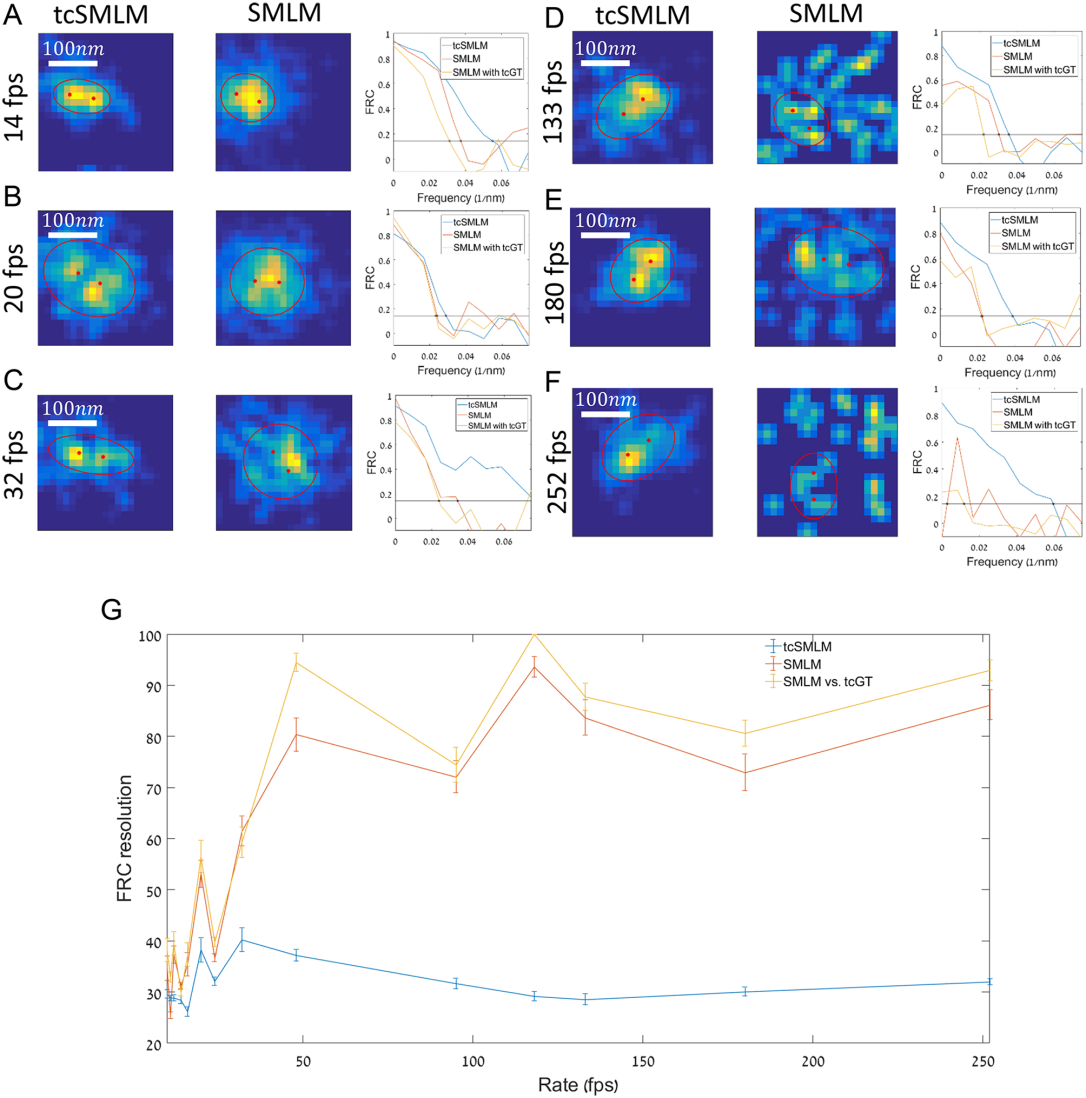
DNA ruler analysis using tcSMLM and SMLM. (**A**) Frame rate = 14fps, zoom image of a representative DNA ruler resolved using tcSMLM (left) and zoom image of the same DNA ruler in SMLM (right). Multiple representative pixels are highlighted in the zoom image for further analysis and comparison below. (**B**–**F**) Same as (**A**), with the respective frame rates: 20 fps, 32 fps, 133 fps, 180 fps and 252 fps. (**G**) Mean FRC resolution dependence on the frame rate (fps). FRC resolution of tcSMLM is shown in blue; FRC resolution of SMLM is shown in red; FRC resolution of SMLM vs. GT_tcSMLM_ is shown in yellow.

We show results from experimental data where the RawData was obtained with following frame rates: 14 fps (Fig. 7A), 20 fps (Fig. 7B), 32 fps (Fig. 7C), 133 fps (Fig. 7D), 180 fps (Fig. 7E) and 252 fps (Fig. 7F). The RawData was then reconstructed using either SMLM or tcSMLM. After reconstruction in either way, we could readily identify the locations of individual nano-ruler particles. To such single nano-rulers, we fitted an ellipse around their corresponding intensity peaks. We then added two points (red points) along the major axis of the ellipse with 25 nm separation of each from the center of the fitted ellipse. These points became representations of the ground truth, as determined by each of the reconstruction methods (namely, GT_SMLM_ and GT_tcSMLM_).

The left panel for each of the representative images in Fig. 7A–F is a zoom image of one tcSMLM nano-ruler, and next to it (to the right) the SMLM zoom image of the same area. Often, after tcSMLM reconstruction we could readily identify two separate peaks ~ 50 nm apart (matching the two ends of the nano-rulers). In contrast, such peaks could not be resolved after SMLM reconstruction, which tended to show a single (effectively merged) peak (Fig. 7A–C middle panels), or a diffused and noisy pattern at relatively high acquisition rates (Fig. 7D–F, middle panels).

We also conducted FRC analysis for each of the identified nano-rulers, throughout our imaging. The rightmost panel shows the FRC of the tcSMLM vs. the GT_tcSMLM_ (Fig. 7A–F, blue line), SMLM vs. the GT_SMLM_ representation for the SMLM (red line) and the FRC of SMLM vs. the GT_tcSMLM_ (yellow line; see Methods for further details). The FRC cut-off was chosen as a default at 1/7. We found that tcSMLM resolution was generally higher than SMLM resolution under all imaging conditions. In the fast frame rates, the poor performance of SMLM in resolving the nano-rulers tends to critically compromise its FRC resolution relative to tcSMLM.

Next, we present the mean FRC resolution as a function of acquisition rate for > 10 nano-rulers at each of the following frame rates: 10, 11, 12, 14, 16, 20, 24, 32, 48, 95, 118, 133, 180 and 152 fps (Fig. 7G). The same locations were chosen for tcSMLM and SMLM (Error bars are SEM). We show that at relatively low frame rates (< 32 fps) tcSMLM shows ~ 20% improvement in FRC resolution, as compared to SMLM (Fig. 7G, compare blue line with orange and yellow lines). Strikingly, for frame rates above 32 fps tcSMLM has an FRC resolution of between two and threefold higher than SMLM.

We conclude that tcSMLM provides significantly better results on experimental data with frame rates above 32 fps, and is most useful when applied to high density samples, in which more than one PSF is acquired at each diffraction-limited area.

We also quantified the performance of tcSMLM reconstruction vs. SMLM reconstruction using simulations of imaged fluorophores at a wide range of acquisition rates (5–2000 fps; Fig. S10). The higher acquisition rates (> 500 fps) may become more applicable and common using state-of-the-art and future sCMOS cameras. We note that the simulations considered relatively negligible noise to allow for useful imaging at such high frame rates. The simulative and experimental results show a similar trend by which tcSMLM shows a superior FRC resolution that becomes more pronounced with acquisition rate, esp. above 50fps (compare Fig. 7G with S10C).

## Discussion

Here we introduce an approach to employ information embedded in temporal correlations of single emitters to enhance SMLM reconstruction. We demonstrate the application of this approach, which we call tcSMLM, to simulations and experimental data. Specifically, we tested a wide range of cases and conditions that included multiple samples, fluorophore types, and publically available datasets. We find that this approach significantly enhances the resolution and the fidelity of SMLM reconstruction when acquiring information at high frame rates. This approach is especially helpful under stringent imaging conditions, such as high density of fluorophores and low SBR, but it is also significantly better under more common imaging conditions for SMLM. The enhanced performance of tcSMLM vs. SMLM reconstruction was demonstrated both experimentally, using DNA nano-rulers, and through extensive simulations. Importantly, we provide guidelines for the optimization of tcSMLM reconstruction for various fluorophores and under the various imaging conditions.

Multiple SMLM reconstruction algorithms have been published^11^. Most of these algorithms rely on the detection of individual emitters in each and every frame separately (or by comparing changes only between two consecutive frames). This approach disregards the information that is embedded in the whole time trajectory of each fluorophore. Other approaches use temporal information of emitters, but do not provide their explicit localization and are limited in resolution in comparison to SMLM. The temporal information has been shown to assist in identifying and localizing individual emitters^8,19,35^. In our approach, we embed the temporal information in the time trajectory of the single molecules in a new dataset, called tcData, thus utilizing the advantages of temporal statistics. This temporal information is provided prior to SMLM reconstruction and thus inherently increases the localization precision. Importantly, while differing from published SMLM algorithms, our approach can serve in tandem to these algorithms for improvement of their performance. For that, one first employs the first steps of tcSMLM reconstruction by building tcData (Fig. 1, items (1)–(5)). Subsequently, our use of thunderSTORM for SMLM reconstruction (as applied to tcData; Fig. 1, generating item (6)) is replaced with an SMLM algorithm of choice (as long as it does not already consider temporal information by itself). Specifically, we demonstrate the performance enhancement of RapidSTORM, RadialSymmetry, DeconSTORM and SRRF.

In our approach, we aimed to allow its wide utility and democratized application. As such, tcSMLM does not require any additional expensive hardware or changes in the SMLM experimental setup. We operated our code in MatlabR2016b on a standard PC (i7 processor, quad core; parallel threading of CPUs was employed with linear acceleration with the number of cores). We also verified its proper operation on multiple other computers and Matlab versions. The code is accessible through Github [https://github.com/ShermanLab/tcSMLM]. Labs proficient in SMLM can simply use tcSMLM with their current system and their choice of reconstruction algorithms. TcSMLM reconstruction takes longer relative to standard SMLM and we have not demonstrated its use for real-time processing of acquired data, as done by others^23,36^. Still, our algorithm can be easily optimized for accelerated operation, esp. by using GPUs^37^.

We have shown the performance of tcSMLM vs. SMLM using both experimental data (Fig. 7) and simulations (Fig. S8; see also Table S1 and SI note 6). We observed that tcSMLM performance becomes esp. better at acquisition rates > 30–50 fps. Still, SMLM acquisition is often performed at lower rates, of 10–20 fps, depending on the fluorophore of choice, the acquisition speed of the camera and additional experimental requirements^38^. Acquisition at high frame rates reduces the number of photons per pixel while increasing read-out noise, and hence often reduces the overall signal to noise ratio (SNR)^21,22^. It may also require limiting the FOV, e.g. by binning of the camera's pixels (Table S2). Hence, on one hand, the faster the rate, the more temporal information can be extracted from intensity trajectories. This is primarily useful for live-cell imaging. On the other hand, raising the acquisition rate also means that the spatial information may be compromised due to the poorer SNR. Practically, capturing RawData with high frame rates can be achieved using common photoswitchable dyes^41^ and fast detectors such as sCMOS cameras^38–40,42^. Thus, tcSMLM may become a useful tool esp. when such means are employed for live cell imaging.

We note a specific limitation to our approach if imaging frame rate is significantly slower than the typical bleaching time of the emitters under study. Such imaging would result in no added information in the time trajectory of the emitters and in more overlapping emitters in the diffraction limited area. In such a case, the performance of tcSMLM would either be comparable to the performance of standard SMLM reconstruction or result in under-counting and artifacts. In contrast, tcSMLM reconstruction naturally benefits from accelerated imaging. Such imaging becomes increasingly accessible through the use of sCMOS detectors^38–40,42^. Also, our study has considered relatively constant or gradually changing densities of fluorophores in the acquired field. Sudden changes in such densities, e.g. due to sudden raising the photoactivation intensity of the fluorophores, may require dynamic changes in the tcSMLM reconstruction parameters. Since this feature has not been included in our algorithm, such abrupt changes may lead to a compromise in the performance of our algorithm. To conclude, we provide here an easy and widely accessible approach for enhancing SMLM reconstruction by employing temporal information of individual emitters. We expect that our approach will be widely adopted for SMLM reconstruction of experimental data.

## Materials and methods

### Cell lines and cloning

Jurkat E6.1 (CD4^+^) T cells were a kind gift from the Samelson lab at the NIH. For the expression of proteins tagged with photoactivatable fluorescent proteins, cells were transfected with the desired DNA plasmid by using NEON electroporator (Invitrogen). Jurkat E6.1 stably expressing TCRζ-Dronpa cell lines were available for this study from previous work^43^. Briefly, cell lines were created by selection with Geneticin at 1.5 mg/ml (G418, Invitrogen). Two–three weeks later, cells were sorted and single clones were grown in 96 well plates. Cells were finally evaluated by flow cytometry, biochemistry assays, confocal microscopy and epifluorescence.

### Sample preparation

#### Staining with Alexa Fluor 647 (anti mouse IgG1)

Standard glass chambers (Ibidi) were coated with $$500\,{\upmu \hbox{l}}$$ of 0.01% poly-l-lysine (diluted in DDW) and left at room temperature for 15 min. The liquid was drained and then dried at 37 °C for 2 h. A reagent of $$6\,{ \upmu \hbox{l}}$$$${\upalpha {\rm CD}}3$$/$$600\,{ \upmu \hbox{l PBS}}$$ was poured into one chamber. The chambers were inserted in the oven at 37 °C for 2 h. The chambers were then washed with PBS five times. Next, a solution of $$1.5\,{ \upmu \hbox{l}}$$ Alexa647/$$1.5\hbox{ ml}$$ was inserted into the chamber and was left there for an hour in room temperature. Finally, we washed the chamber 5 times with PBS and $$620\,{ \upmu \hbox{l}}$$ Buffer B with $$70\,{ \upmu \hbox{l}}$$ MEA and $$7\,{ \upmu \hbox{l}}$$ glucose oxidase (GO) were used as the microscopy buffer suitable for dSTORM imaging.

#### Samples with GP41-PAmCherry and TCRζ-Dronpa

Jurkat T cells transfected with TCRζ-Dronpa and GP41-PAmCherry were suspended in imaging buffer (RPMI without phenol red, 10% FBS, 25 mM Hepes) at a concentration of $\sim \frac{{10}^{6}}{150}\,{\upmu \hbox{l}}$ and ${10}^{5}{-}5\times {10}^{5}$ cells were dropped into chambers coated as described above for the experiment with Alexa647, incubated at 37 °C for 3–5 min, and fixed with 2.4% PFA for 30 min at 37 °C. The chambers were then washed three times with PBS.

#### Samples with LFA–Alexa647

LFA1 proteins were labelled in fixed Jurkat E6.1cells. Here, $5\times {10}^{5}$ cells were incubated for 60 min at RT suspended in a 0.5 µg primary antibody of mouse anti-human CD11a (LFA-1; BD Pharmingen, 555378), diluted in 2% normal goat serum in PFN. This was followed by washing with PFN. Alexa647 was added using a labelled secondary antibody conjugated to Goat anti-Mouse IgG2a (A21241, Life Technologies) diluted (1/3000) in 2% normal goat serum in PFN. Cells were then incubated for 45 min at RT and washed 3 times with PFN.

#### DNA nano-ruler—dSTORM

We purchased STORM 50R DNA nano-rulers from GATTAquant GmbH. We prepared the DNA nano-rulers following the vendor's instructions: Ibidi chambers were washed three times with $500 \,{\upmu \hbox{l}}$ PBS, and then incubated with $200 \,{\upmu \hbox{l}}$ of BSA-biotin solution for $5 \hbox{min}$. Next, we washed the chambers with PBS, and incubated them with $200 \,{\upmu \hbox{l}}$ of neutravidin solution for $5$ min. We then washed the chambers with PBS supplemented with 10 $\hbox{mM}$ magnesium chloride ($1x$ IB: immobilization buffer). For creating the sample, we diluted 1 $\upmu$l of the DNA origami solution with 200 $\upmu$l 1×IB. The chambers were incubated for another $5$ min and then washed three times with 500 $\,{\upmu \hbox{l}}$ of 1×IB. Finally, we used $620\,{ \upmu \hbox{l}}$ Buffer B with $70\,{ \upmu \hbox{l}}$ MEA and $7\,{ \upmu \hbox{l}}$ glucose oxidase (GO) as the microscopy buffer, which is suitable for dSTORM imaging. According to the vendor, each end of the nano-ruler may carry 3–4 Alex647 fluorophores. Thus, some variability in the intensity at the nano-ruler ends is expected.

#### The microscopy system

We performed dSTORM Imaging using a TIRF microscope (Nikon). The excitation of Alexa 647 was done with 647 nm laser. We excited Dronpa with 488 nm laser line. The excitation of PAmCherry was achieved using 561 nm laser line with constant, 3% activation of 405 nm laser. The emission was acquired using iXon^+^ Ultra EMCCD camera (Andor). We used a range of frame rates for acquisition of 32-201fps. Detector pixel size was 16 $\upmu {\hbox{m}}^{2}$ and using an × 100 microscope objective for magnification resulted in de facto pixel size of 160 nm.

### Data processing

We used thunderSTORM as our reconstruction algorithm of choice. Through this software, there are multiple parameters and different PSF models that may be applied. The default parameters were shown to provide the fastest and best results^24^. We tested this set of parameters with the same conclusion. Thus, we used the following general parameters throughout the work: Image filtering wavelet filter (B-Spline) with B-Spline order 3 and scale 2. Approximate localization method of Local maximum with 8-neighbourhood connectivity. For the sub-pixel localization of molecules, we used the Gaussian method, also based on our mathematical background (see SI, note 2). We changed the fitting radius and initial sigma based on the specification of the simulation and experimental data. Multi-emitter fitting analysis was used for the high density simulations in Fig. 4 and Fig. S9 with default parameters.

In Figs. 2, 3, 7, S5, S6 and S9 we presented the reconstructed SMLM images and compared them to the corresponding tcSMLM images. In these cases, we used Gaussian rendering with lateral uncertainty of 1–2 nm, depending on the datasets. We checked other rendering reconstructions and found insignificant changes in the results.

Merging conditions and camera parameters were changed according to the analysed data. For example, the pixel-size was mostly 160 nm as we preferred to use the simulation with conditions similar to our microscopy setup. We chose the gain and camera base-level based on similar considerations. Importantly, we meticulously chose specific grouping conditions for each different dataset, either simulated or experimental. For our simulations, we chose the merged conditions such that we reach the closest possible localization table to the original GT (note that we also used FRC and CBC to find that). Only then, we ran the simulations over the range of parameters, with relevant change to the grouping for each iteration. Also, for our experimental data we used specific parameters, as follows. We changed gradually the off-frames and density parameters, and at the same time examined the super resolved image contours compared to the wide field contours. We chose the parameters that minimally affected these contours. For reconstructing samples with Dronpa, Alexa647 and PamCherry we used the following parameters: off-frames 20, 65 and 25, and grouping distances of 60 nm, 80 nm and 85 nm respectively. Unless explicitly mentioned otherwise in the main text, all results presented in this work were reconstructed after grouping.

Notably, for reasons of consistency, we used the same reconstruction parameters for SMLM and tcSMLM alike, in each of our analyses. We acknowledge that modification of merge parameters (i.e. grouping over space and time) may yield more optimal results for SMLM and tcSMLM reconstructions under the various conditions. Optimal merge parameters for tcSMLM relative to SMLM are provided in the tcData guide (see SI, note 6).

For obtaining the data in Fig. 7, we initially chose peak locations in the final reconstructed super-resolved image (i.e. after either tcSMLM or SMLM reconstruction). These peaks corresponded to the positions of individual nano-rulers. Next, we cut 20 × 20 pixels from the original reconstructed image, roughly around the center of mass (266.6 × 266.$9\, {\hbox{nm}}^{2}$). We then used the Matlab function '*regionprops*' to fit an ellipse around the peaks in the image (Fig. 7A–F). We chose fitting to an ellipse shape since the nano-rulers tend to generally form elongated PSFs that cannot always be separated into 2 point-like PSFs, separated by 50 nm. Next, we defined two points, with 50 nm separation along the major axis of the ellipse and with symmetric placement around the ellipse center (chosen as the axis origin). Those two points were chosen to represent the ground-truth (GTs) for the individual nano-ruler markers. Notably, this GT is not absolute since it only relates to the distance between the two ends of the nano-rulers, but it does not determine the center position of the nano-rulers or their orientation in 2D. Thus, we could define two prospective GTs: GT_SMLM_ and GT_tcSMLM_ by fitting the ellipse to either the SMLM-reconstructed image or to the tcSMLM-reconstructed image, respectively. These GT's (GT_SMLM_ and GT_tcSMLM_) were generally not in agreement and thus, both served for resolution estimation via FRC.

For each acquisition frame rate, we randomly chose 10 nano-ruler locations, and did the same analysis for each location. Then, we calculated an FRC curve for each of these nano-ruler locations vs. their respective GT's (GT_SMLM_ and GT_tcSMLM_).

We observed that tcSMLM reconstruction could detect more accurately the (50 nm-separated) two-ends of the nano-rulers, while these were often merged after SMLM reconstruction; e.g. Fig. 7A,B). If the two ends were essentially unresolved (as often occurred for SMLM at high acquisition rates, and rarely for tcSMLM; e.g. Fig. 7E,F), we set the FRC resolution limit artificially to 100 nm. Therefore, this limit is *conservative* for the resolution enhancement of tcSMLM, as compared to SMLM reconstruction.

For the SMLM location, we also calculated the FRC vs. the GT_tcSMLM_. This latter curves of SMLM vs. GT_tcSMLM_ (Fig. 7, all curves in yellow) can be regarded as *relatively favorable* estimations for tcSMLM resolution enhancement over SMLM. Likewise, the curves of SMLM vs. GT_SMLM_ (Fig. 7, all curves in orange) represent the *least* favorable possible resolution enhancement by tcSMLM over SMLM.

Finally, we chose the mean of the FRC resolution for the 10 points as the reported resolution. Error-bars are SEM.

In Fig. S9 we also present the performance of tcSMLM, combined with the following algorithms: RapidSTORM^44^, RadialSymmetry^45^, DeconSTORM^16^ and SRRF^23^. Each of these published algorithms includes various fitting parameters. Thus, we do not provide their detailed presentation here. Briefly, we entered the basic parameters such as pixel-size, sigma, FWHM and camera parameters according to the specific datasets. As we used a specific simulation in the figure (see main text and Fig. S9), we tried to fine-tune the algorithm parameters toward reconstructing the closest results to the GT. We recognize that optimal parameters of these algorithms should provide better agreement with the GT. However, in tuning the parameters of these algorithms, we realized that as we get better results, the difference between the FRC resolution of tcData localization and of RawData widens. For example, for RapidSTORM, we started with FRC resolution of 92 nm and 105 nm, for tcSMLM and SMLM respectively. However, a better tuning of the algorithm parameters provided 63 nm vs. 84 nm, respectively (or percentage wise: $\sim 12\,{\%}$ vs. $\sim 25\,{\%}$). A similar outcome was observed for the other presented algorithms.

Finally, experimental data contains interferences in their intensity trajectories from various sources of noise and background. In order to find a clear cut-off between PI and PII (see Fig. 6), we smoothed the decay curves using a moving average with a moving-window of size 5–15, depending on the level of noise in the data. We acknowledge that other RawData sets and different averaging moving-windows might optimize the results further. A detailed description along with additional examples is found in our tcData guide (see SI, note 6).

### Simulations

We used the SOFI simulation tool^25^ for the simulations in this study. We also employed additional simulations, either provided by thunderSTORM tools or open-source^11^ with similar results.

Throughout this research we used the following simulation parameters: (1) camera parameters. Readout noise = 1.6 rms, dark current = 0.06 electrons/pixel/s, quantum efficiency = 0.7, gain = 6, pixel size = 16.5×16.5 $\upmu {\hbox{m}}^{2}$; (2) optical parameters. Numerical aperture = 1.3, wavelength = 600 nm and magnification = 100. Other parameters were changed in accordance with the specific analysis. Changes in the default parameters typically require changes in the parameters of analyses, either in the reconstruction algorithm or in the FRC/CBC parameters; but the results remained the same under such changes. We used the Siemens star pattern to avoid anisotropic interferences in the spatial frequencies that could affect the FRC results.

In Figs. 4A–H, 5C,D and 7C,D, each point in the graph is made of 5 simulations. Each of these individual simulation had identical set of parameters, except for the locations of the emitters, as they were randomly scattered across the same Siemens star. Thus, each point represents the mean of the results for those simulations, and the error is the standard error of the mean (SEM). We chose 5 simulations for simplicity, and further found that a higher number of simulations did not change the error significantly.

## Supplementary information

Supplementary information.

## Data Availability

The authors declare that the data supporting the findings of this study are available within the article and its supplementary information files, or are available upon reasonable requests to the authors. In addition, we provide a GitHub link to the scripts, examples and guide: https://github.com/ShermanLab/tcSMLM.

## References

[1] Betzig E (2006). Imaging intracellular fluorescent proteins at nanometer resolution. Science.

[2] Hess ST, Girirajan TPK, Mason MD (2006). Ultra-high resolution imaging by fluorescence photoactivation localization microscopy. Biophys. J..

[3] Rust MJ, Bates M, Zhuang X (2006). Sub-diffraction-limit imaging by stochastic optical reconstruction microscopy (STORM). Nat. Methods.

[4] Heilemann M (2008). Subdiffraction-resolution fluorescence imaging with conventional fluorescent probes. Angew. Chem. Int. Ed..

[5] Dickson RM, Cubittt AB, Tsient RY, Moerner WE (1997). On/off blinking and switching behaviour of single molecules of green fluorescent protein. Nature.

[6] Heilemann M, Margeat E, Kasper R, Sauer M, Tinnefeld P (2005). Carbocyanine dyes as efficient reversible single-molecule optical switch. J. Am. Chem. Soc..

[7] Dempsey GT, Vaughan JC, Chen KH, Bates M, Zhuang X (2011). Evaluation of fluorophores for optimal performance in localization-based super-resolution imaging. Nat. Methods.

[8] Lee SH, Shin JY, Lee A, Bustamante C (2012). Counting single photoactivatable fluorescent molecules by photoactivated localization microscopy (PALM). Proc. Natl. Acad. Sci. U. S. A..

[9] Nieuwenhuizen RPJ (2013). Measuring image resolution in optical nanoscopy. Nat. Methods.

[10] Sage D (2015). Quantitative evaluation of software packages for single-molecule localization microscopy. Nat. Methods.

[11] Sage D (2019). Super-resolution fight club: assessment of 2D and 3D single-molecule localization microscopy software. Nat. Methods.

[12] Dertinger T, Colyer R, Iyer G, Weiss S, Enderlein J (2009). Fast, background-free, 3D super-resolution optical fluctuation imaging (SOFI). Proc. Natl. Acad. Sci. U. S. A..

[13] Agarwal K, Machá R (2016). Multiple signal classification algorithm for super-resolution fluorescence microscopy. Nat. Commun..

[14] Solomon O, Mutzafi M, Segev M, Eldar YC (2018). Sparsity-based super-resolution microscopy from correlation information: erratum. Opt. Express.

[15] Cox S (2012). Bayesian localization microscopy reveals nanoscale podosome dynamics. Nat. Methods.

[16] Mukamel EA, Babcock H, Zhuang X (2012). Statistical deconvolution for superresolution fluorescence microscopy. Biophys. J..

[17] Burnette DT, Sengupta P, Dai Y, Lippincott-Schwartz J, Kachar B (2011). Bleaching/blinking assisted localization microscopy for superresolution imaging using standard fluorescent molecules. Proc. Natl. Acad. Sci. U. S. A..

[18] Marsh RJ (2018). Artifact-free high-density localization microscopy analysis. Nat. Methods.

[19] Deschout H (2016). Complementarity of PALM and SOFI for super-resolution live-cell imaging of focal adhesions. Nat. Commun..

[20] Schidorsky S (2018). Synergizing superresolution optical fluctuation imaging with single molecule localization microscopy. Methods Appl. Fluoresc..

[21] Annibale P, Vanni S, Scarselli M, Rothlisberger U, Radenovic A (2011). Identification of clustering artifacts in photoactivated localization microscopy. Nat. Methods.

[22] Flors C (2007). A stroboscopic approach for fast photoactivation-localization microscopy with Dronpa mutants. J. Am. Chem. Soc..

[23] Gustafsson N (2016). Fast live-cell conventional fluorophore nanoscopy with ImageJ through super-resolution radial fluctuations. Nat. Commun..

[24] Ovesný M, Křížek P, Borkovec J, Švindrych Z, Hagen GM (2014). ThunderSTORM: A comprehensive ImageJ plug-in for PALM and STORM data analysis and super-resolution imaging. Bioinformatics.

[25] Girsault A (2016). SOFI simulation tool: a software package for simulating and testing super-resolution optical fluctuation imaging. PLoS ONE.

[26] Panchuk-Voloshina N (1999). Alexa dyes, a series of new fluorescent dyes that yield exceptionally bright, photostable conjugates. J. Histochem. Cytochem..

[27] Manley S, Gunzenhäuser J, Olivier N (2011). A starter kit for point-localization super-resolution imaging. Curr. Opin. Chem. Biol..

[28] Van Heel M (1987). Similarity measures between images. Ultramicroscopy.

[29] Malkusch S (2012). Coordinate-based colocalization analysis of single-molecule localization microscopy data. Histochem. Cell Biol..

[30] Dertinger T, Colyer R, Iyer G, Weiss S, Enderlein J (2009). fluctuation imaging (SOFI). Proc. Natl. Acad. Sci. U. S. A..

[31] Bates M, Huang B, Dempsey GT, Zhuang X (2007). Multicolor super-resolution imaging with photo-switchable fluorescent probes. Science (80-).

[32] Rosten E, Jones GE, Cox S (2013). ImageJ plug-in for Bayesian analysis of blinking and bleaching. Nat. Methods.

[33] Hendrix J, Flors C, Dedecker P, Hofkens J, Engelborghs Y (2008). Dark states in monomeric red fluorescent proteins studied by fluorescence correlation and single molecule spectroscopy. Biophys. J..

[34] Schmied JJ (2014). DNA origami-based standards for quantitative fluorescence microscopy. Nat. Protoc..

[35] Dertinger T, Colyer R, Vogel R, Enderlein J, Weiss S (2010). Achieving increased resolution and more pixels with Superresolution Optical Fluctuation Imaging (SOFI). Opt. Express.

[36] Kechkar A, Nair D, Heilemann M, Choquet D, Sibarita JB (2013). Real-time analysis and visualization for single-molecule based super-resolution microscopy. PLoS ONE.

[37] Barrachina S (2009). Exploiting the capabilities of modern GPUs for dense matrix computations. Concurr. Comput. Pract. Exp..

[38] Diekmann R (2017). Characterization of an industry-grade CMOS camera well suited for single molecule localization microscopy—high performance super-resolution at low cost. Sci. Rep..

[39] Quan T, Zeng S, Huang Z-L (2012). Errata: Localization capability and limitation of electron-multiplying charge-coupled, scientific complementary metal-oxide semiconductor, and charge-coupled devices for superresolution imaging. J. Biomed. Opt..

[40] Huang Z-L (2011). Localization-based super-resolution microscopy with an sCMOS camera. Opt. Express.

[41] Jones SA, Shim SH, He J, Zhuang X (2011). Fast, three-dimensional super-resolution imaging of live cells. Nat. Methods.

[42] Huang F (2013). Video-rate nanoscopy using sCMOS camera-specific single-molecule localization algorithms. Nat. Methods.

[43] Sherman E (2011). Functional nanoscale organization of signaling molecules downstream of the T cell antigen receptor. Immunity.

[44] Wolter S (2012). RapidSTORM: accurate, fast open-source software for localization microscopy. Nat. Methods.

[45] Parthasarathy R (2012). Rapid, accurate particle tracking by calculation of radial symmetry centers. Nat. Methods.

