# Supplementary material for: Time-correlated single molecule localization microscopy enhances resolution and fidelity
Source: Sci Rep. 2020 Oct 1;10:16212. doi: 10.1038/s41598-020-72812-y (PMC7529757; doi:10.1038/s41598-020-72812-y)
Supplement: Supplementary file 1 — Supplementary information. [file 41598_2020_72812_MOESM1_ESM.docx]

Time-correlated single molecule localization microscopy enhances resolution and fidelity

Kobi Hermon^1^, Shachar Schidorsky^1^, Yair Razvag^1^, Oren Yakovian^1^, Eilon Sherman^1^*

^1^Racah Institute of Physics, The Hebrew University, Jerusalem, Israel, 91904

* Corresponding author

**Supplementary Information**

mail: [eilon.sherman@mail.huji.ac.il](mailto:eilon.sherman@mail.huji.ac.il)

**Supplementary Table**

**Table S1. Simulations emulating experimental data & experimental data values**

| **Simulation**  **Number**  **in Fig. S8** | **Density (active per** $\boldsymbol{\mu}\boldsymbol{m}^{\boldsymbol{2}}$**)** | **SBR / Average SBR** | **On state (ms)** | **Off state (ms)** | **Decay time (ms)** | **Bleaching time (s)** | **Acquisition time (s)** | **Acquisition rate (fps)** |
| --- | --- | --- | --- | --- | --- | --- | --- | --- |
| **(*)** | 10 | 10 | 36 | 3600 | ~35 | 6 | 30 | 250 |
| **(1)** | 10 | 20 | 470 | 1000 | 320 | 2 | 30 | 250 |
| **(2)** | 10 | 7 | 353 | 500 | 207 | 4 | 30 | 250 |
| **(3)** | 10 | 6 | 100 | 180 | 65 | 4 | 30 | 250 |
| **(4)** | 15 | 4 | 110 | 180 | 68 | 6 | 30 | 250 |
| **Experimental data** | | | **In figs:** | |  | | | |
| **(1)** | ~4 | ~20 | Fig. 2C | | ~320 | ~2 | 10 | 50 |
| **(2)** | ~6 | ~7 | Fig. S9A | | ~207 | **~**4 | 18 | 100 |
| **(3)** | ~10 | ~6 | Fig. 2G | | ~65 | ~3.6 | 75 | 25 |
| **(4)** | ~15 | ~4 | Fig. 2E | | ~68 | ~6.8 | 120 | 25 |

**Table S2: FOV size (in pixels) as a function of acquisition rate**

| **ROI / FOV (pixels)** | **Frame rate (fps)*** |
| --- | --- |
| $250x250$ | 103 |
| $200x200$ | 128 |
| $150x150$ | 166 |
| $120x120$ | 199 |
| $100x100$ | 232 |
| $50x50$ | 385 |
| $25x25$ | 594 |

**Comments:**

* Imaging performed using iXon^+^ Ultra EMCCD camera (Andor); Gain = 250; Readout: 17MHz, 16bit mode

**Supplementary figures and legends**


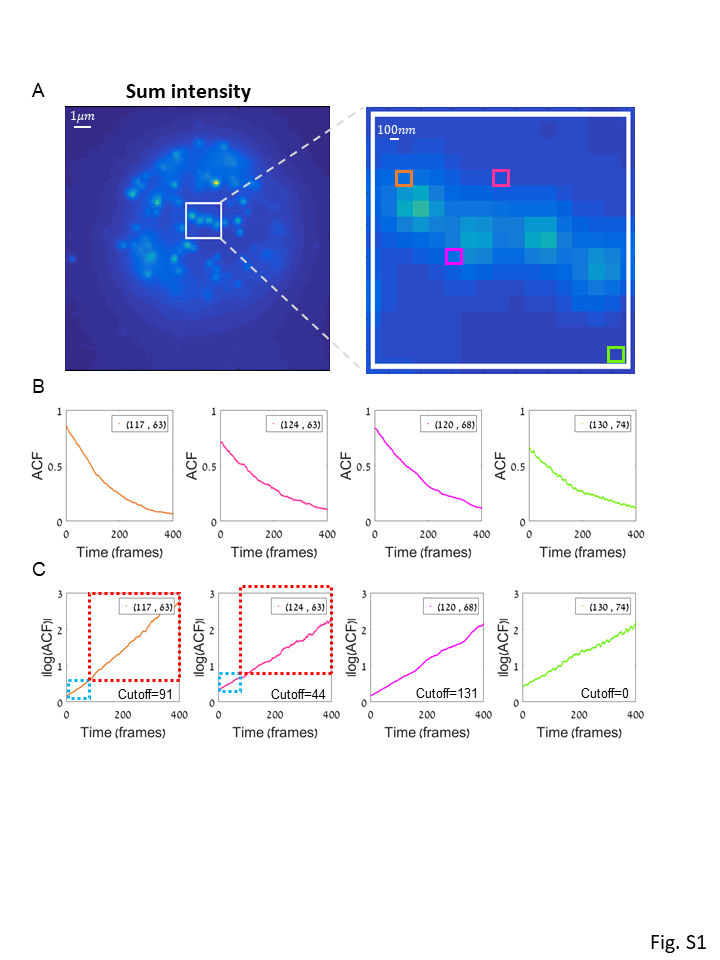


**Fig. S1. Demonstrating the finding of optimal moving window on LFA labelled with Alexa647**

(A) Jurkat E6.1 cells were labelled with an αLFA antibody labelled with Alexa647 and imaged with an acquisition rate of 55.6 fps. Shown are the sum intensity of one cell (left) and zoom images (right). Multiple representative pixels are highlighted in the zoom image for further analysis and comparison below. (B) The auto-correlation of the pixels highlighted in zoom image in A. PI and PII in B,C,D, blue and red dotted areas, stand for the ACF decay of one emitter vs. total trajectory decay (see main text). (C) The absolute value of the log(auto-correlation) of the pixels highlighted in zoom image in A. (D) The slope of the absolute value of the log(auto-correlation) of the pixels highlighted in zoom image in A.


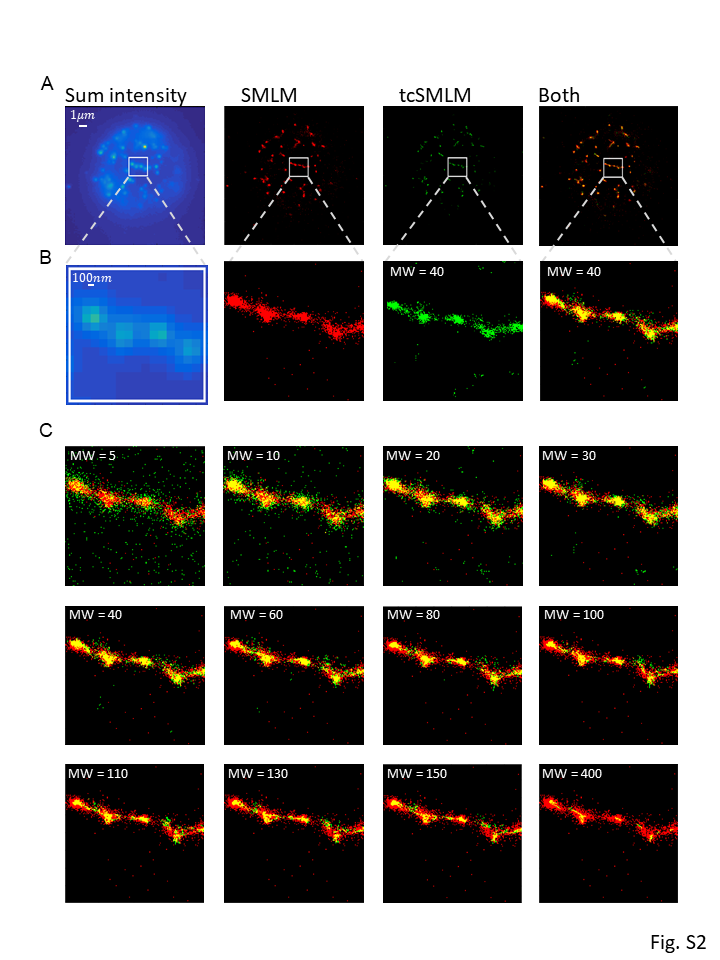


**Fig. S2. The dependence of *tcSMLM* resolution enhancement on reconstruction window size for LFA labelled with Alexa647**

(A) Jurkat E6.1 cells were labelled with an αLFA antibody labelled with Alexa647 and imaged. Shown are the sum intensity of one cell (left) and its SMLM and tcSMLM reconstructions, and the overlay of SMLM (red) and tcSMLM (green) reconstructions. Corresponding zoom images are shown at the bottom row.

(B) The overlay of SMLM (red) and tcSMLM (green) reconstructions of the data in A (zoom images) using moving windows of 5, 10, 20, 30, 40, 60, 80, 100, 110, 130, 150 and 400 frames.

**
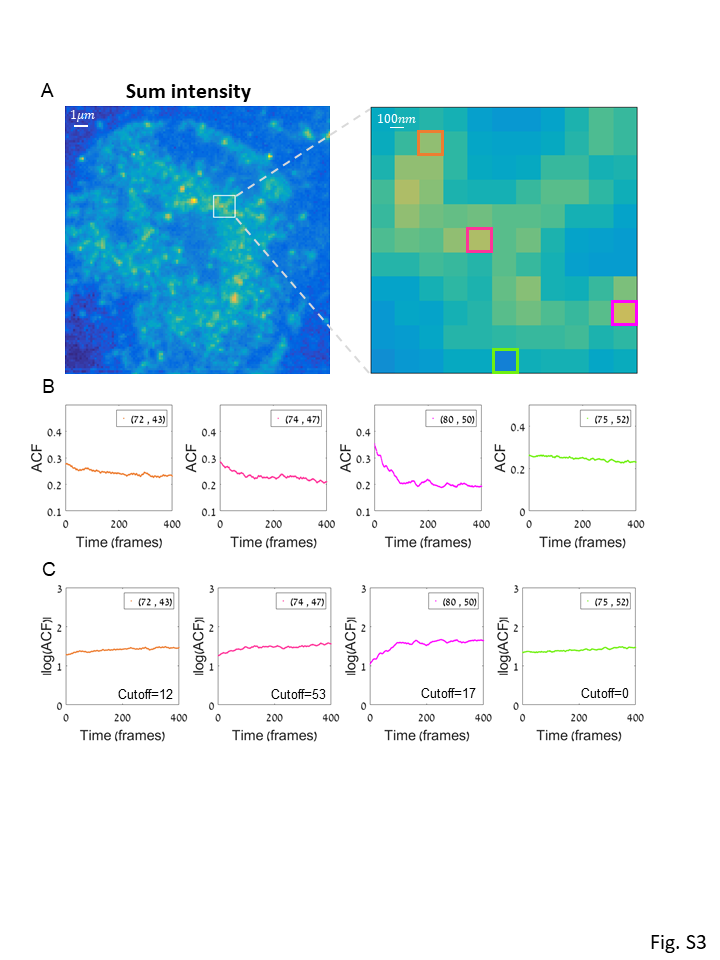
**

**Fig. S3. Demonstrating the effect of high frame rate on finding the optimal moving window for TCR**$\boldsymbol{\zeta}$**-Dronpa**

(A) Jurkat E6.1 cells expressing TCRζ-Dronpa were imaged. Shown are the sum intensity of one cell (left) and zoom images (right). Multiple representative pixels are highlighted in the zoom image for further analysis and comparison below. (B) The auto-correlation of the pixels highlighted in zoom image in A. PI and PII in B,C,D, blue and red dotted areas, stand for the ACF decay of one emitter vs. total trajectory decay (see main text). (C) The absolute value of the log(auto-correlation) of the pixels highlighted in zoom image in A. (D) The slope of the absolute value of the log(auto-correlation) of the pixels highlighted in zoom image in A.

**
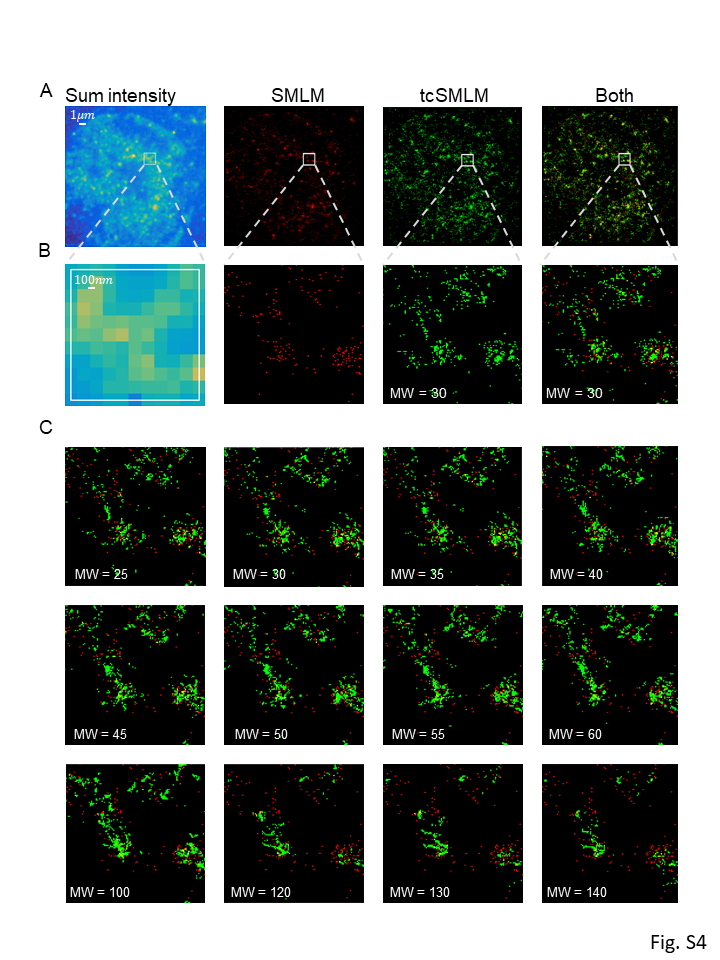
**

**Fig. S4. The dependence of *tcSMLM* resolution enhancement on reconstruction window-size for TCR**$\boldsymbol{\zeta}$**-Dronpa**

(A) Jurkat E6.1 cells expressing TCRζ-Dronpa were imaged. Shown are the sum intensity of one cell (top row, left) and its SMLM and tcSMLM reconstructions, and the overlay of SMLM (red) and tcSMLM (green) reconstructions. Corresponding zoom images are shown at the bottom row. (B) The overlay of SMLM (red) and tcSMLM (green) reconstructions of the data in A (zoom images) using moving windows of 25, 30, 35, 40, 45, 50, 55, 60, 100, 120, 130, and 140 frames.

**
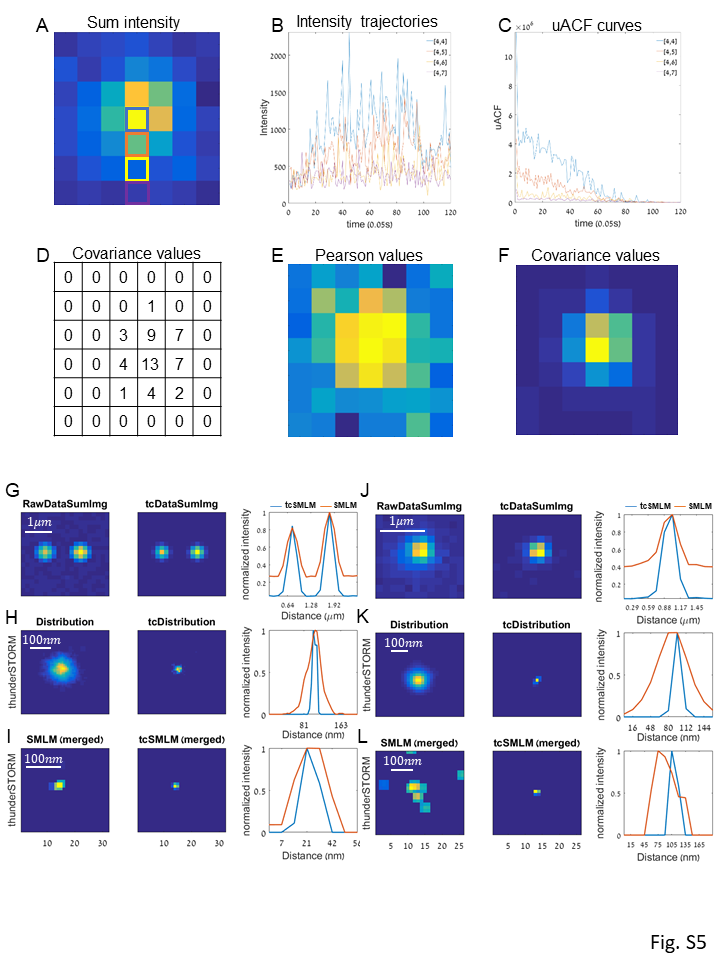
**

**Fig. S5. Demonstrating the effect of the PSF enhancement for tcData and tcSMLM**

(A-F) The use of Cov for tcSMLM analyses. (A) The sum intensity of a single Alexaflour 647 is presented (experimental data). (B) Intensity fluctuation of specified pixels are shown. The pixels with the blue and orange frames contain signal, while the pixels with the yellow and purple frames contain mostly noise. (C) The uACF curves of the intensity trajectories in B. (D) Table of the Covariance values (as compared to the ‘averaged’ model). The values are rounded for simplicity. (E) Pearson correlation values presented as an image. (F) Covariance values presented as an image. (G-I) tcSMLM vs SMLM reconstruction for simulated data. (G) A simulation of two emitters is presented. Shown are the sum intensity of RawData and tcData, andthe intensity profile along the center-line for both (top row, right). (H) The localization distribution for SMLM and tcSMLM is shown (before merging) as well as the intensity profile along the center-line for both. (I) The localization distribution for SMLM and tcSMLM is shown after merging as well as the intensity profile along the center-line for both. (J-L) tcSMLM vs SMLM reconstruction for experimental data. (J) The sum intensity of a single Alexafluor 647. simulation of two emitters is presented. Presented are the RawData and tcData, and the intensity profile along the center-line for both (top row, right). (K) The localization distribution for SMLM and tcSMLM is shown for the data in panel D before merging, as well as the intensity profile along the center-line for both. (L) The localization distribution for SMLM and tcSMLM is shown after merging for the data in panel D, as well as the intensity profile along the center-line for both.

**
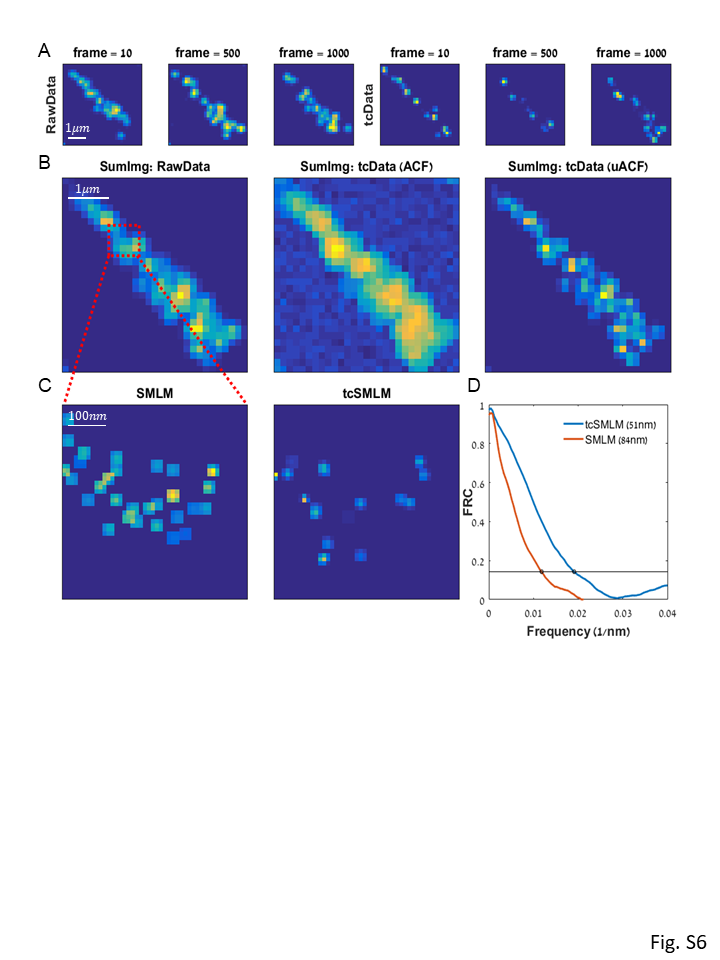
**

**Fig. S6. Reconstruction enhancement of mildly overlapping emitters**

(A) A series of 3 frames from the RawData movie (left) and tcData movie (right). The frames presented are: 10, 500 and 1000. (B) The SumImg of RawData (left), ‘tcData’ created with ACF (middle) and tcData created with uACF (right) are shown. (C) A segment of SMLM reconstruction (left) tcSMLM (middle). (D) FRC analysis for the the zoom in B for SMLM (ornage) and tcSMLM (blue) reconstruction, as compared to GT (right), with resulted resolution is 84nm and 51nm, respectively.


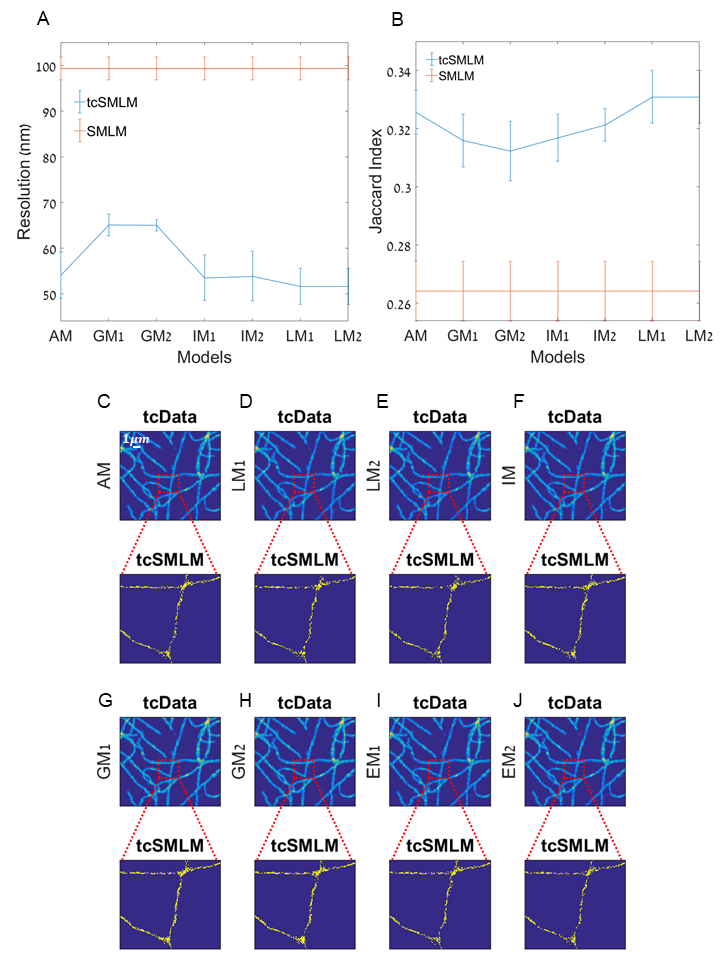


**Fig. S7. Different fluorophore models and their impact on FRC resolution and the Jaccard Index**

(A) FRC resolution vs. 7 different fluorophore models for SMLM (red) and tcSMLM (blue). These models are described in Supplementary Note 5. (B) Jaccard Index vs. 7 different fluorophore models for SMLM (red) and tcSMLM (blue). Models are as in panel A. (C) Published data of dSTORM imaging of Alexa Fluor 647 labelled antibodies scattered on a coverslip coated with PLL (top), and zoom image after reconstruction with tcSMLM (bottom) using the Averaged Model (AM). (D) Same as C. with Linear Model1 (LM1). (E) Same as C. with Linear Model2 (LM2). (F) Same as C. with “Ideal Model” (IM). (G) Same as C. with Gaussian Model1 (GM1). (H) Same as C. with Gaussian Model2 (GM2). (I) Same as C. with Exponential Model1 (EM1). (J) Same as C. with Exponential Model2 (EM2).


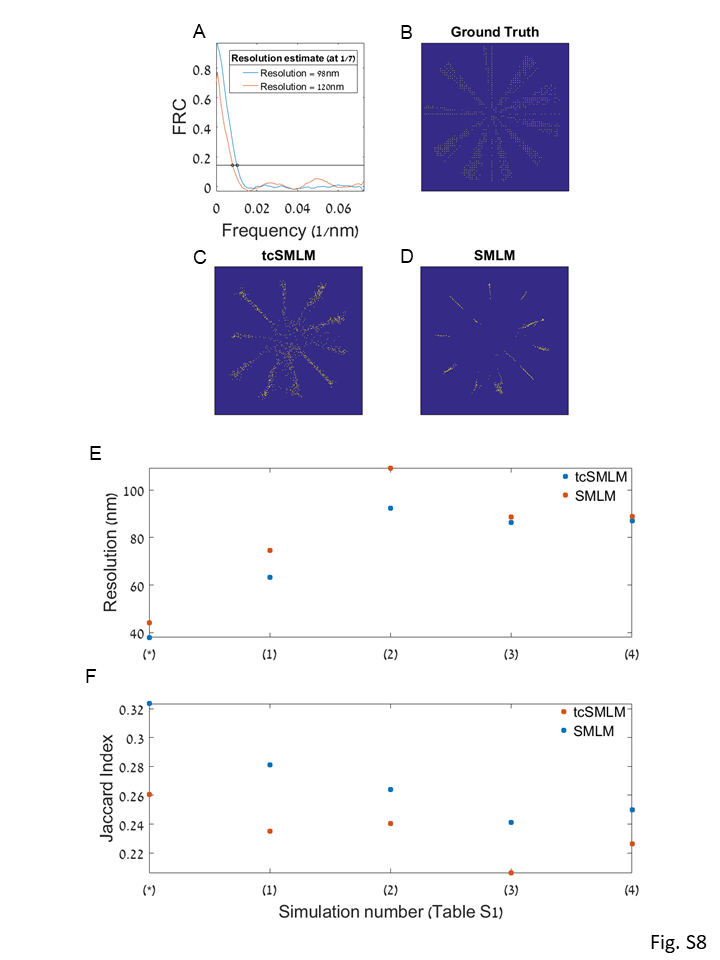


**Fig. S8. Simulation with various photo-physical parameters**

(A) FRC resolution for a simulation similar to the one in Fig. 3, yet with 10 times more active emitters per $\mu m^{2}$. (B) The GT for the high density simulation. (C) and (D) are the super-resolved images reconstructed by either tcSMLM or SMLM, respectively. (E) FRC resolution for multiple simulations [marked as either (*) or (1)-(4), and as detailed in Table. S1]. Shown are results for tcSMLM (blue discs) and SMLM (red discs). (F) Jaccard Index values for the simulation (*) and (1)-(4) presented in Table. S1. Blue discs represent tcSMLM and red discs represent SMLM.


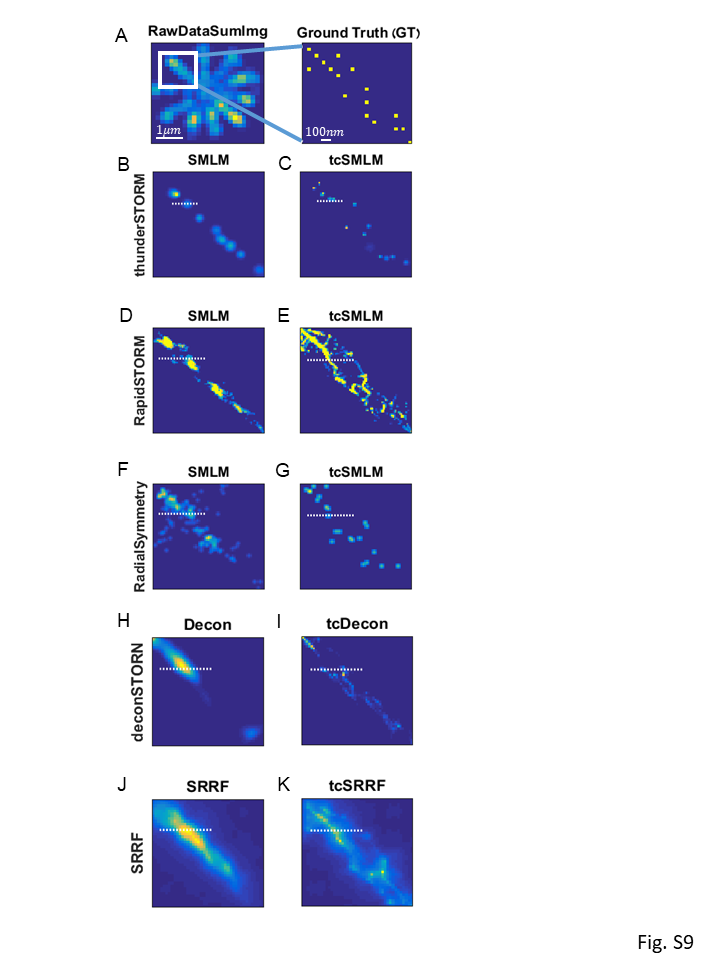


**Fig. S9. Synergic application of tcSMLM with additional SMLM and SR reconstruction algorithms**

(A) Simulated data of fluorophores embedded in a Siemens star (left) and zoom image (right). (B) Zoom image, as in A, after SMLM reconstruction using thunderSTORM. (C) Zoom image, as in A, after tcSMLM reconstruction using thunderstorm. (D-K) similar images as in panels B-C, when using either only RapidSTORM reconstruction, or RapidSTORM on tcData. (F-G) similar images as in panels B-C, when using either only RadialSymmetry reconstruction, or RadialSymmetry on tcData. (H-I) similar images and analyses as in panels B-B, when using either only DeconSTORM reconstruction, or DeconSTORM on tcData. (J-K) similar images and analyses as in panels B-C, when using either only SRRF reconstruction, or SRRF on tcData. Artificial sharpening by the different reconstruction techniques in panels B-K cannot be ruled out.


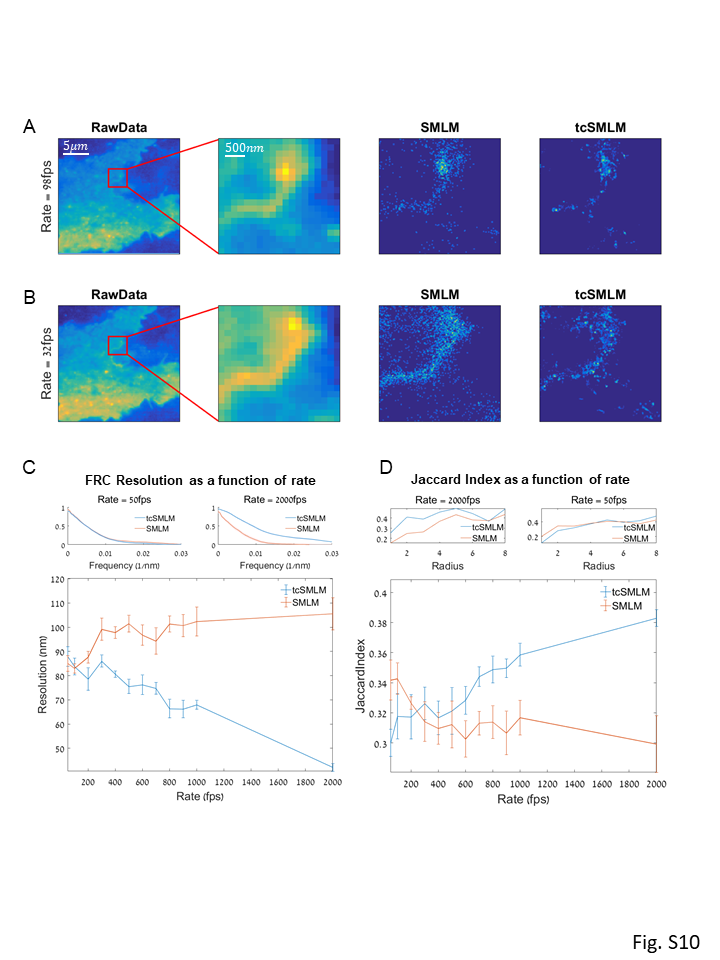


**Fig. S10. tcSMLM enhancement as a function of imaging frame rate**

(A) Images of a PM region of A375 melanoma cell expressing PAmCherry-NRAS and acquired at a frame rate of 98 fps. A zoom, SMLM- and tcSMLM-reconstructed images are shown on right. (B). Images of a PM region of A375 melanoma cell expressing PAmCherry-NRAS and acquired at a frame rate of 32 fps. A zoom, SMLM- and tcSMLM-reconstructed images are shown on right. (C) The FRC resolution of SMLM (orange) and tcSMLM (blue) reconstructions as a function of the acquisition rate. (D) The Jaccard Index of SMLM (orange) and tcSMLM (blue) reconstructions as a function of the acquisition rate.

**Supplemental Notes**

**Supplementary note 1 – Finding the optimal moving window for LFA-AF647 and reconstruction performance**

We imaged E6.1 cells where LFA membrane molecules were conjugated to a primary αLFA antibody and a secondary antibody, labelled with Alexa flour 647. The LFA molecules were imaged via dSTORM (Figs. S1, S2). We used a frame rate of 55 fps and acquired 3000 frames per cell.

Equivalent to Fig. 6 in our main text, we zoomed on a distinctive area in the 'sumimg' for each *RawData* and chose multiple representative pixels (Fig. S1A). We show here 4 locations representing different levels of intensity in the 'sumimg' (Fig. S1A: green, orange, red and purple squares). Importantly, to validate our approach we chose one pixel resembling noise (i.e. having a very low summed intensity). According to our method (See main text Fig. 6), we expected the cut off to be $\sim0$ (Fig. S1A, zoomed segment, green pixel).

We performed $ACF$ over the full intensity trajectory of each location. Here, we show only a part of the trajectory (400 frames), to emphasize the difference between PI and PII (Fig. S1 B; For further details, see main text, Fig. 6B). Next, we studied the $log (ACF)$ curves. The smoothed exponential decay part transforms to a linear line, while the rest of the curve shows the changes in the ACF decay (Fig. S1C).

Finally, in order to find the exact cut-off from *PI* to *PII*, we looked at the derivative of $Log(ACF)$ (Fig. S1, D). For a linear function, meaning along the PI, we should get an approximated parallel line to the time axis. The time values for which the curve deviates from the parallel line correspond to the point in time when PI changes to PII. We received a unique time value for each location. In order to capture most emitters, we chose MW as: $MW=min(cutoff\left( PI\left( n \right) \right))$, where *n* represents the number of locations chosen (e.g., 4 locations in these examples).

In conclusion, the appropriate MW we found, based on this analysis, was $MW\approx44$ for LFA, labelled with Alexa Fluor 647 (AF647)**.** Moreover, the representative noise-only-pixel (Fig. S1, A, zoomed segment, green pixels) indeed resulted with cutoff = 0.

Next, we studied the dependence of tcSMLM resolution enhancement on reconstruction window size for Alexa647-LFA. Similar to Fig. 5 in our main text, we demonstrate here (Fig. S2), visually, the effect of the MW size on experimental data (Same as in Fig. S1). For that, we recorded the tcSMLM and SMLM images for MW = 5, 10, 20, 30, 40, 60, 80, 100, 110, 130, 150 and 400. We merged these images to show the relative performance of these reconstruction algorithms (Fig. S2C). The results of tcSMLM are shown in green and SMLM in red.

Initially, we show the sum intensity of the experimental data, along a full reconstructed image of tcSMLM and SMLM for MW = 40 (Fig. S2A). To each image, a magnified segment is attached (Fig. S2B). The last image for panels A and B is the merged image of tcSMLM and SMLM.

We further show the gradual change in the relative performance of tcSMLM to SMLM. We see that for MW in the range of 20-40 the results vary slowly, which adheres to the results shown in Fig. 7 in our main text, and present a reasonable margin of error. Moreover, tcSMLM mostly appear in the centre of the reconstructed SMLM and is more concentrated and less noisy. These results indicate a better resolving capacity, as shown in the simulations presented in Figs. 3,4.

Finally, tcSMLM shows less counts using larger MWs. This is supported by the theory presented in the main text of Fig. 6. We observe more counts for smaller MWs, as *tcSMLM* captures the out-of-focus noise which is correlated to the internal photo-physics of the fluorophores.

**Supplementary note 2 - Demonstrating the effect of high frame rate on finding the optimal moving window for** $\boldsymbol{\zeta}$**-Dronpa and reconstruction performance**

We imaged Jurkat cells expressing TCR$\zeta$-Dronpa via PALM (Fig. S3A). Here, we used Dronpa, a green photoactivatable fluorescent protein (PAFP)^1^. Dronpa is known for having a relatively fast bleaching time after excitation by a $488nm$ laser^1^. Intentionally, we used the highest frame rate our camera is capable of for ROIs of 124X124 pixels. These conditions allowed an acquisition at $200fps$. We acquired 1500 frames (7.5 sec) for each cell. At such a rate, the signal per pixel is roughly $\frac{1}{5}$ of our previously used rate of $40fps$. Therefore, this imaging was more sensitive to out of focus noise, and thus with lower SBR. The ratio of signal to background greatly impacts SMLM localization precision; the lower it is, the poorer the precision^2^. Thus, under these stringent conditions, common SMLM algorithms are less useful.

In order to use tcSMLM optimally, we needed to find the appropriate MW to create the tcData. However, with the low SBR in the acquired data of this experiment, the PI curve (Fig. S3B; Also see main text, Fig. 6) is very bumpy as the intensity trajectory includes substantial out of focus interferences. As such, utilizing our approach to find the MW was less efficient (Fig. S3C). Here, for example, we found an optimal MW of ~12, which is too low for extracting temporal information (Fig. S3D).

Empirically and using simulations, we found that in such cases where we get low MWs the optimal MW range is 25-40. Here, we chose MW=30, and hereinafter (Fig. S4) we show that any choice within that range seems to provide better results than SMLM.

Next, we studied the dependence of tcSMLM resolution enhancement on reconstruction window-size for the TCR$\zeta$-Dronpa data. Here (Fig. S4; Same Exp. Data as in Fig. S3), we recorded the tcSMLM and SMLM images for MW = 25, 30, 35, 40, 45, 50, 55, 60, 100, 120, 130 and 140. We merged these images to show the relative performance of these reconstruction algorithms (Fig. S4C). The results of *tcSMLM* are shown in green and SMLM in red.

Initially, we show the sum intensity of the experimental data, along with a reconstructed image of tcSMLM and SMLM for MW = 30 (Fig. S4A). To each image, a corresponding zoom image is shown (Fig. S4B). The last image for panels A and B is the merged image of tcSMLM and SMLM.

We clearly see that tcSMLM reconstructed more emitters and is showing more fidelity to the contours of the unresolved data (Fig. S4A,B). Moreover, the tcSMLM reconstructed images are less noisy outside the visible pattern of protein at the cell membrane. Generally, tcSMLM was able to reconstruct denser data, clearly featuring super-resolved information of the original image (Fig. S4B).

We further show the gradual change in the performance of tcSMLM, relative to SMLM. We see that for MWs in the range of 30-50, the tcSMLM results are very similar. As we use larger MWs, bypassing the highest MW found in Fig. S3D (MW=53), the resulted tcData had less unique fluorophore information and more average trajectory information. Therefore, we see significant changes in the results and less reconstructed data.

Finally, tcSMLM was able to reconstruct a better resolved image with higher fidelity in the example, while SMLM was generally unable to resolve the information hidden in the intensity trajectories. Importantly, a high acquisition rate of 200fps has fifth of the amount of data relative to acquisition at 40fps, and for the same number of frames. We conclude that tcSMLM is able to reconstruct the image with significantly less information, and therefore, can potentially extract data from fast processes in vivo.

**Supplemental note 3 - A mathematical background for the algorithm**

*3.1 Background*

Consider a blinking fluorophore in a sample under certain imaging conditions. The specific emission dynamics arise from transitions between energy levels of electronic states. Thus, the fluorophore emits photons in a series of on- and off-times ^3^, termed $\tau_{i}$. The emission continues until it reaches a long-lived dark state or until it is irreversibly photo-bleached. We refer to this fluorescence extinction time as $\tau_{B}$. Fluorophores from the same species may have both static and dynamic heterogeneities and thus, may have wide variability in their on- and off-times ^456^.

The emission of a fluorophore is captured by a sensor through its point-spread function (PSF). The PSF typically extends over multiple (say, $NxN$) pixels in the sensor, and is often approximated as a Gaussian distribution, with a standard deviation of σ ^7^. The intensity in each of these pixels can be described in a time-trajectory vector with unique temporal behavior. Ideally, the autocorrelation function (ACF) for a time trajectory decays exponentially as a function of the total decay time, $\tau_{T}$ that incorporates the on-off times. Defining $k_{i}=\frac{1}{\tau_{i}}$, the sum $k_{T}=\sum_{i=1}^{n} k_{i}$, were n represents the total number of independent processes with distinguishable on-off times, we find $\tau_{T}=\frac{1}{K_{T}}$. In order to extract $\tau_{T}$ of a fluorophore, we need a specific moving window (MW) of the time trajectory, such that: $MW<T_{B}$. If $T_{B}$ is very long, the size of MW is limited only imaging conditions. Otherwise for a short $T_{B}$, the possibility to extract $\tau_{T}$ is limited by Nyquist criterion. Hence the importance of frame rate: faster fps lead to optimal MW choice.

Practically, an imaging system is never ideal. There are noise factors, and in dense samples, emitters overlap either in the diffraction limited area or/and temporally in the same time-trajectory. As a result, the MW part of the time-trajectory might include more than one fluorophore. Moreover, out-of-focus noise may possess the same intensity behavior as a real fluorophore, and therefore impact the decay time of the ACF. Thus, the practical use of $\tau_{T}$ is limited. Here, we perform Covariance (Cov) between ACF over MWs of time trajectories to a unique fluorophore model, effectively correlating all time trajectories to a single model. Instead of using standard ACF, we use an un-normalized autocorrelation function (uACF). The use of uACF provides a continued distinction between different MWs of the time trajectory. Otherwise, the distinctions are blended and the result will be blurred and ineffective (Fig. S6B).

Let us start with a distinguishable single fluorophore in a sample. We assume that the emitters are uncorrelated, and thus their intensity temporal trajectories are independent and are stationary for the MW time. Under such assumptions, it has been shown for SOFI^8^ that performing a second order correlation function (equivalent to the 2^nd^ order cumulant) over the PSF yields a new set of data with an effective PSF raised to the second power: $PSF^{2}$. Since we begin our approach with a slightly different function, we detail below its related mathematical considerations as well. For simplicity, we follow here the same mathematical notation as for SOFI (elaborated thoroughly in ^9^). Starting with the mathematical description of a single emitter:

$F\left( r,t \right)=U\left( r_{i} \right)f_{i}(t)$ (1)

where $U(r_{i})$ is the system PSF, *r* is the position and *i* refers to a specific emitter. $f_{i}(t)$ refers to the time trajectory starting at t=0 and ending in the time length of MW, $\tau_{MW}$. $f_{i}(t)$ could be written as:

$f_{i}\left( t \right)=\boldsymbol{\epsilon}_{i}s_{i}(t)$ (2)

where $\epsilon_{i}$ describes the brightness and $s_{i}(t)$ represents binary temporal fluctuations in the intensity of the fluorophore. Here, we use uACF. Using (1) and (2) we get:

$$uACF\left( F\left( r,\tau\right) \right)=E\left( \delta F\left( r,t \right)\delta F\left( r, t+\tau\right) \right)$$

$=$ $U^{2}\left( r \right)\boldsymbol{\epsilon}_{i}^{2}E\left( \delta s_{i}\left( t \right)\delta s_{i}\left( t+\tau\right) \right)$ (3)

where *E(X)* is the expected value of *X* when averaged over t, and where $\delta X\left( r,t \right)=X-E(X)$. For simplicity, the description is for a single emitter and for a single time-lag. Thus, Eq. 3 shows that for uACF, we effectively get: $PSF^{2}$. We return to this point in section 3.3 below.

*3.2 Employing the Covariance*

As of now, we have one set of uACF curves, with a certain decay time encapsulating the internal photo-physics of the related fluorophore. However, what we need is a single value for each trajectory. One way to obtain such a value is through the correlation of the uACF curves with a fluorophore model. Essentially, we want to evaluate the variations (compared to the mean) in each curve vs. the variations in the model. The common approach to calculate such correlations is using the Pearson correlation ^10^. However, we note an issue with employing the Pearson correlation ‘as is’ for this goal.

The reason is that trajectories emitted from the same fluorophore have the same (i.e. correlated) variations. Therefore, their Pearson coefficient will be the same. Thus, the outcome of the Pearson correlation would yield a flat spatial distribution rather than the spatial PSF of a single emitter (see Fig. S5F), which resembles a Gaussian distribution.

To preserve the Gaussian distribution for the tcSMLM process, we use the un-normalized Pearson correlation coefficient, which is simply the Covariance. Let us now define a time-trajectory model for the fluorophore as: $m_{i}\left( t \right)=\boldsymbol{\epsilon}_{\boldsymbol{i}}^{'}s_{i}^{'}(t)$. Thus, for the moving window (MW), located at a specific frame in rawData, one can compute one tcData frame as:

$$tcData(r,r^{'})=Cov\left( uACF\left( F\left( r,\tau\right) \right),uACF\left( m\left( \tau\right) \right) \right)=$$

$$=Cov\left( U^{2}\left( r \right)\boldsymbol{\epsilon}_{i}^{2}E\left( \delta s_{i}\left( t \right)\delta s_{i}\left( t+\tau\right) \right),\boldsymbol{\epsilon}_{i}^{'2}E\left( \delta s_{i}^{'}\left( t \right)\delta s_{i}^{'}\left( t+\tau\right) \right) \right)=$$

$=U^{2}\left( r \right)\boldsymbol{\epsilon}_{i}^{2}\boldsymbol{\epsilon}_{i}^{'2}Cov\left( E\left( \delta s_{i}\left( t \right)\delta s_{i}\left( t+\tau\right) \right),E\left( \delta s_{i}^{'}\left( t \right)\delta s_{i}^{'}\left( t+\tau\right) \right) \right)$ (4)

where $Cov\left( E\left( \delta s_{i}\left( t \right)\delta s_{i}\left( t+\tau\right) \right),E\left( \delta s_{i}^{'}\left( t \right)\delta s_{i}^{'}\left( t+\tau\right) \right) \right)$ is the covariance performed over the time axis of the moving window. This operation in Eq. 4 is then performed at multiple time-points in RawData, yielding the complete tcData(r,t).

Based on Eq. 4, we the conclude following: if the uACF of the model and the time trajectory are uncorrelated, the covariance is 0, clearing the tcData frame further from noise. The same goes for out-of-focus noise from multiple uncorrelated emitters (see Fig. S5D,F), which varies significantly compared to the model. Otherwise, the level of variation is represented as the covariance value (see Fig. S5A,B).

*3.3 PSF narrowing and noise reduction*

We suggest in our approach an ‘averaged’ model. This yields an averaged uACF over a moving window as chosen for the analysis of tcSMLM. This model is updated with the shift of the MW during the analyses (see Fig. 1). Empirically, the averaged model performs similar to the fluorophore model (capturing the ideal fluorophore photophysics), and it is thus used throughout the research. As we approximate the PSF in the new data (tcData) with a Gaussian distribution, we effectively reduce the original standard deviation by a factor of $\sqrt{2}$:

$std=\frac{\sigma}{\sqrt{2}}$ (5)

This simply follows the definition of the Gaussian and Eq. 4 (for n = 2):

$N\left( \mu,\sigma^{2} \right)=a\cdot e^{-\frac{\left( x-b \right)^{2}}{2\sigma^{2}}}\Longrightarrow{GN\left( \mu,\sigma^{2} \right)}^{n}\left( x \right)=a^{n}\cdot e^{-\frac{\left( x-b \right)^{2}}{(\left( \frac{2}{\sqrt{n}} \right){\sigma)}^{2}}}\Longrightarrow\sigma_{new}=\frac{\sigma}{\sqrt{n}}$

Thus, each frame in the tcData, same as SOFI order 2, is super resolved at least by a factor of $\sqrt{2}$.

Notably, we employ here various localization algorithms over tcData. The physical limit of localization precision is defined by the Cramer-Rao criterion ^11^. A criterion that includes background noise, pixilation noise and counting noise is ^2^:

$\left\langle\left( \Delta x \right)^{2} \right\rangle=\frac{s^{2}+\frac{a^{2}}{12}}{N}+\frac{4\sqrt{\pi}s^{3}b^{2}}{aN^{2}}.$

Based on Eq. 5, we can also write:

$$\left\langle\left( \Delta x' \right)^{2} \right\rangle=\frac{\left( \frac{s}{\sqrt{2}} \right)^{2}+\frac{a^{2}}{12}}{N}+\frac{4\sqrt{\pi}\left( \frac{s}{\sqrt{2}} \right)^{3}b^{2}}{aN^{2}} <\left\langle\left( \Delta x \right)^{2} \right\rangle(6)$$

The uncorrelated noise factors in the data acquired from an imaging system, are cleared as a direct result of applying the uACF over the time trajectory (see section 3.1). Additionally, the covariance operation reduces further the uncorrelated data that may remain in the uACF curve, as it is being correlated with a model. Such uncorrelated data may include background noise from multiple emitters that varies (compared to its mean) differently than the model. Therefore, the ‘b’ factor in Eq. 6 is effectively descreased. As a result, in the localization process the precision enhancement surpass the limit described in Eq. 6 (Fig. S5I,L).

*3.4 Further considerations for single molecule localization*

Post processing methods are based on additional information provided by the data and the system under study. Techniques such as drift correction ^12^ and removal of duplicates ^13^ are regularly used to improve the accuracy and precision of single molecule localization. However, finding the optimal merging parameters for experimental data is a non-trivial task that typically involves some arbitrary choices. The improvement of tcSMLM in precision can potentially be as good as localization with grouping (Fig. S5H,K). As such, it provides a way to avoid errors in the single molecule analysis (see also main text and Fig. 2C,F,I,L).

Finally, there are some conditions for using tcSMLM effectively. Firstly, if the temporal resolution is longer than MW, for sparse data with high SNR tcSMLM is either better or equivalent to SMLM (Fig. S5H,I). Under the same temporal resolution but under stringent imaging conditions, tcSMLM provides higher resolution, better SNR and consequently, better fidelity (see main text and Fig. 4). However, in cases where there is only partial temporal separation between neighboring fluorophores, it becomes more complicated. If the temporal resolution is minimal, i.e. it is good enough for SMLM reconstruction but significantly lower than the MW time-length, the ability to enhance results by tcSMLM would be insufficient. The reason is as follows: tcData essentially include data from all MW frames. Therefore, at low temporal resolution, the enhancement in SNR and resolution in tcData comes at the expense of higher number of overlapping emitters in the same diffraction-limited area. However, in cases where the temporal separation is very low there is typically a high density of emitters, and thus more than one emitter in the diffraction limited zone. The pros of tcSMLM in such cases significantly overcomes the marginal loss of spatial sparsity, as the improvement of resolution in tcData results in higher separation in the diffraction-limited area and the reduction of noise provides better localization (Fig. S6A,C). Lastly, for samples with extremely high density of fluorophores, that is where SMLM is ineffective, tcSMLM performs slightly better than SMLM in resolution and fidelity (see main text and Fig. 4F,H). Our work is focused mainly on providing a thorough understanding of tcSMLM pros and cons.

**Supplementary note 4 - Demonstrating the tcData and tcSMLM enhancment (Eqs. 5,6)**

To demonstrate the use of the covariance coefficient as a correlation tool, we scattered antibodies labelled with *Alexa647 flour* (AF647) on a field of view (see Methods) and chose a single representative region of interest (Fig. S5A). The intensity in this region originates from either a single emitter, and is distributed following the emitter’s PSF. We present here four intensity trajectories that match individual pixels (Fig. S5B) and their uACF curves (Fig. S5C). Using the process mentioned above, we determined which trajectory represents an emitter and which is noise. We show here a table of the covariance values of the curves vs. the averaged model, as calculated for a single frame of tcData following the procedure in Note 3 (Fig. S5D; for simplicity we rounded the resultant Cov values). Next, we show the difference between the values of the covariance and the Pearson coefficient (Fig. S5E,F). These results show a higher contrast of the PSF and better noise reduction for the covariance outcome (Fig. S5F) relative to the Pearson correlation (Fig. S5F). This clarifies our choice of using the covariance over the Pearson correlation.

Next, we simulated two emitters under standard conditions for both SMLM and tcSMLM, with the exception of high frame rate of 1000fps. The emitters were well outside the diffraction-limited area of each other at a distance of 640nm. First, we show both the sum intensity of the RawData and, next to it, the sum ‘intensity’ of tcData (Fig. S5G). Next, we present a segment of the center line for both sum intensities, representing the center line of both emitters (Fig. S5G, far right plot). The FWHM for tcData PSF is smaller than the RawData PSF in a factor of $\sim\frac{1}{1.71}.$

In addition, we show the localization distribution before grouping, while showing the same segment through the center-line of the localization presented (Fig. S5H). Here, the FWHM of the tcDisribution PSF is smaller than the FHDM of the RawData distribution PSF by a factor of $\sim\frac{1}{2.6}$. The enhancement in precision is larger than theory due to less random noise in tcData, added to the resolution enhancement of the original PSF. Finally, as the details of the simulation are known a-priori, the localizations with grouping converge to the same resolution of tcSMLM and SMLM for the simplified case of a single emitter (Fig. S5I).

Apart from the simulation, we show an representative PSF of Alexa flour 647 (Fig. S5D; same experiment as Fig. S5A). The imaging conditions were approximately equal to the conditions of the simulation as we defined the simulation parameters according to the experimental data. The FHWM of the tcData PSF is smaller than the RawData PSF by a factor of $\sim\frac{1}{2}$ (Fig. S5D, right panel). Thus, the enhancement is significantly higher than theorized in Eq. 5. In addition, for the distribution of localizations, the FWHM of the tcSMLM PSF is smaller than the FHDM of the RawData distribution PSF by a factor of $\sim\frac{1}{4.33}$. This again greatly improves the enhancement in addition to the narrowing of the PSF. Finally, as the data here is experimental, merging parameters are somewhat arbitrarily chosen based on the original dataset. Here, the resolution of the tcSMLM final localization is significantly better as compared to SMLM by a factor of $\sim2.4$ (Fig. S5F, segmented plot). Choosing a wide range of other merging parameters did not change the result significantly. We attribute this to the noise in the RawData that distorted the PSF, as compared to the noise reduction in the tcData.

Moreover, we created a simulation with mildly overlapping emitters, having long bleaching times (Fig. S6). That is, normally more than three emitters in the same diffraction-limited area and throughout the whole movie. Such conditions provide low temporal resolution and stringent conditions for standard SMLM. This case is closely related to relatively sparse imaging of fluorescence emitters in a regular microscopic system. First, we present a series of frames for RawData (Fig. S6A, left) and tcData (Fig. S6A, right). Next, we examine the SumImg of the RawData, as compared to the SumImg of ‘tcData’ created with ACF function (u-tcData) and SumImg of standard tcData created with uACF (Fig. S6B). We observe that the ‘u-tcData’ is blurred, the SNR is lower and the distinction between individual emitters is worsened compared to the RawData. The tcData, however, shows better distinction between individual emitters and clear-cut contours of the sample (Fig. S6B, far right). Finally, we reconstructed the SMLM and tcSMLM from the RawData and tcData sets (Fig. S6C). We used multi-emitter fitting of thunderstorm for SMLM with the number of emitters in each diffraction-limited area equal to 3. For tcData, however, we used regular conditions and didn’t use multi-emitter fitting. SMLM was not able to reconstruct the set properly and yielded relatively poor resolution under a wide range of parameters, in addition to the one presented here, which is the best of them ($84nm$). In contrast, tcSMLM reconstructed the data set with an FRC resolution of $51nm$, regardless of changes in the merging parameters (Fig. S6C, right plot).

**Supplementary note 5 – The impact of different fluorophore models on simulated and experimental data**

In Fig. S7 we used simulation as in Fig. 3 with lower SBR, and 4000 frames. We created (arbitrarily) 6 new models: Linear Model1 (LM1): $y\left( t \right)=t$; Linear Model2 (LM2): $y\left( t \right)=5t+12$. The Gaussian model was created using the ‘wgn’ Matlab add-on: Gaussian Model1 (GM1): wgn(4000,1,1); Gaussian Model2 (GM2): wgn(4000,1,500); Exponential Model1 (EM1): $f\left( t \right)=e^{t}+5$; Exponential Model2 (EM2): $f\left( t \right)=e^{\frac{1}{5}t}+10$. We name as Ideal models (IM1) pixel trajectories representing a fluorophore for simulated or experimental data.

The FRC resolution vs. 7 models, among them the averaged model (AM) suggested as a default, shows significant differences only in using a random Gaussian models (Fig. S7A). The Jaccard Index shows the same results (Fig. S7B). Next, we used Published data of dSTORM imaging of Alexa Fluor 647 labelled antibodies scattered on a coverslip coated with PLL (Fig. S7C-J). We show the tcData sumimg for 8 models (Fig. S7C-J, top) and zoom image of their tcSMLM reconstruction (Fig. S7C-J, bottom). The results are generally similar, while the averaged model performs almost equally to the ideal model. We conclude that the choice of the model does not change significantly the tcSMLM results and while any model could work, the averaged model (AM) performs well and can serve as a good default choice.

**Supplementary note 6 - Simulations resembling experimental results and very dense simulation**

First, we present the same simulation as in Fig.3A with 10 times the number of active emitters per $\mu m^{2}$ (Fig. S8A). Note that the GT is enlarged (by 12-fold) in order to show the emitters better. Under such high density conditions, both tcSMLM and SMLM performs poorly, yet tcSMLM shows a 20% increase of resolution in comparison to SMLM (Fig. S8A), and the results look much closer to GT (compare Fig. S8C,D with Fig. S8B).

Next, we present photophysical values of 4 different simulations, in tandem with 4 experimental results (In Table S1). We used density values where more than 3 active emitters were present in a diffraction limited spot. Such conditions highlight the advantage of tcSMLM in these conditions (see Fig. S6 and Supp. note 4). We also chose an acquisition rate of 250fps for all simulations, which is higher than commonly used. Such rates are naturally beneficial for tcSMLM reconstruction. For lower acquisition rates (up to $\sim50fps$), tcSMLM still has an advantage over SMLM, yet it is less significant. The resolution and Jaccard Index values for each simulation, including the high density simulation, are presented in Fig. S8E,F respectively. We conclude that for experimental data, as long as tcSMLM conditions are kept (decay time lower than bleaching time; high enough acquisition rate), tcSMLM performs better in terms of resolution and fidelity. This occurs under conditions of both relatively high and low (active) fluorophore densities.

**Supplementary note 7 - Using tcSMLM with additional SMLM reconstruction algorithms**

We claim that tcData can be analysed as in tcSMLM using additional (and likely, any) SMLM reconstruction algorithms that do not already consider temporal fluctuations in the intensity of fluorophores. Moreover, we claim that tcSMLM has an advantage over regular SMLM for high acquisition rates, and that it can be especially useful under conditions of high density, poor temporal resolution and low SBR. Recently, advanced localization algorithms were developed showing better reconstruction performance under dense conditions. Among them are DeconSTORM ^14^ and SRRF ^15^. We show in Fig. 8 that applying these algorithms over tcData provide enhanced results using a dense simulation (Fig. S9, as in Fig. 7). In addition, we show the results of applying representative SMLM algorithms, namely RapidSTORM ^16^ and RadialSymmetry ^17^ over tcData in comparison to RawData.

Here, we used a simulation with high frame rate of $1000fps$ (Fig. S9A), where tcSMLM is significantly advantageous (see Fig. 7). Other parameters as in Fig. 3. Indeed, thunderSTORM, RapidSTORM, RadialSymmetry, DeconSTORM and SRRF show, visually, very low fidelity to the dense simulation (Fig. S9B,D,F,H,J). However, once applied over tcData, the contours of the simulated pattern are more clearly defined (Fig. 8C,E,G,I,K). In addition, for the SMLM algorithms thunderSTORM, RapidSTORM and RadialSymmetry, the separation of individual emitters is significantly better using tcData (Fig. 8C,F,G).

We term employing SRRF on tcData - 'tcSRRF', and employing DeconSTORM on tcData - 'tcDecon'.

**Supplementary note 8 - The effect of fast frame rate on tcSMLM**

We have shown the performance of tcSMLM with varied frame rates for DNA nan rulers (Fig. 7) where the highest frame rate was 252fps. The results were significantly better for the faster frame rates. In other figures, we chose simulations resembling fast acquisition frame rates, $500fps$ and above. Here, we show experimental data of real cells as well as simulations. In order to evaluate the performance of tcSMLM at relatively fast frames rates, we chose simulation conditions with negligible noise.

First, we show qualitative results from experimental data where the RawData was obtained with two frame rates: 98fps (Fig. S10A) and 32fps (Fig. S10B). Here, we imaged the plasma membrane of A375 melanoma cells, expressing PAmCherry-NRas (Fig. S10A,B). The same cells were imaged with a range of frame rates and for a fixed number of frames. As such, the amount of acquired data for the lower frame rate was higher. For the higher frame rate (Fig. S10A), the uncertainty median was $40nm$ for SMLM, compared to $34nm$ for tcSMLM. For the lower frame rate (Fig. S10B), we found that the uncertainty median for SMLM was $47nm$, compared to $40nm$ for tcSMLM. While the tcSMLM results are lower in both cases, the change is more significant for the higher frame rate. Taken together with the data presented previously (Fig. 2D-J), tcSMLM also shows a reconstructed image that is less noisy and with more distinctive details (Fig. S10A,B; compare two rightmost zoom images in each panel).

Next, we present the FRC resolution and JI as a function of acquisition rate for simulations resembling frame rates of 50-1000fps (Figs. S10C,D). Importantly, we chose a simulation with temporally overlapping emitters, for which common SMLM reconstruction algorithms are less efficient (same as in Fig. 3 and Fig. 7). We used a density of $\sim20$ active emitters per $\mu m^{2}$ and negligible noise. We show that tcSMLM performs significantly better for frame rates above 200fps, while at lower frame rates SMLM and tcSMLM achieve similar resolution while tcSMLM has slightly (and insignificantly) lower fidelity (Figs. 7C,D). Notably, the resolution enhancement and fidelity for tcSMLM significantly increases with the frame rate, while for SMLM the resolution remains relatively unchanged, the same as shown in Fig. 7G (Fig. S10C). We conclude, as in the experimental nano-rulers (Fig. 7G), that tcSMLM provides significantly better results for data acquired with fast acquisition rates, and is most useful when applied to high density samples, in which more than one PSF is acquired at each diffraction-limited area. In negligible noise, for frame rates lower than 50fps, SMLM performs either similar or insignificantly better than tcSMLM.

**Supplementary note 9 – User's guideline for installing and operating tcSMLM**

In the GitHub link [<https://github.com/ShermanLab/tcSMLM>], there is a zip folder named tcData. Inside there are matlab files and a few examples – simulations and experimental data. The pdf file named “tcDataInstructions” contain a guide showing the use of 2 specific examples. Following the guide is the easiest way to get acquainted with tcData.

**Supplemental References**

1. Ando, R., Mizuno, H. & Miyawaki, A. Regulated fast nucleocytoplasmic shuttling observed by reversible protein highlighting. *Science (80-. ).* **306,** 1370–1373 (2004).

2. Thompson, R. E., Larson, D. R. & Webb, W. W. Precise nanometer localization analysis for individual fluorescent probes. *Biophys. J.* **82,** 2775–83 (2002).

3. Dickson, R. M., Cubittt, A. B., Tsient, R. Y. & Moerner, W. E. On/off blinking and switching behaviour of single molecules of green fluorescent protein. *Nature* **388,** 355–358 (1997).

4. Vogelsang, J. *et al.* A reducing and oxidizing system minimizes photobleaching and blinking of fluorescent dyes. *Angew. Chemie - Int. Ed.* **47,** 5465–5469 (2008).

5. Flors, C. *et al.* A stroboscopic approach for fast photoactivation-localization microscopy with Dronpa mutants. *J. Am. Chem. Soc.* **129,** 13970–13977 (2007).

6. Stennett, E. M. S., Ciuba, M. A. & Levitus, M. Photophysical processes in single molecule organic fluorescent probes. *Chemical Society Reviews* **43,** 1057–1075 (2014).

7. Zhang, B., Zerubia, J. & Olivo-Marin, J. C. Gaussian approximations of fluorescence microscope point-spread function models. in *Applied Optics* **46,** 1819–1829 (2007).

8. Dertinger, T., Colyer, R., Iyer, G., Weiss, S. & Enderlein, J. Fast, background-free, 3D super-resolution optical fluctuation imaging (SOFI). *Proc. Natl. Acad. Sci. U. S. A.* **106,** 22287–92 (2009).

9. Dertinger, T., Colyer, R., Vogel, R., Enderlein, J. & Weiss, S. Achieving increased resolution and more pixels with Superresolution Optical Fluctuation Imaging (SOFI). *Opt. Express* **18,** 18875 (2010).

10. Adler, J. & Parmryd, I. Quantifying colocalization by correlation: The pearson correlation coefficient is superior to the Mander’s overlap coefficient. *Cytom. Part A* **77,** 733–742 (2010).

11. Winick, K. A. Cramér–Rao lower bounds on the performance of charge-coupled-device optical position estimators. *J. Opt. Soc. Am. A* **3,** 1809 (1986).

12. Mlodzianoski, M. J. *et al.* Sample drift correction in 3D fluorescence photoactivation localization microscopy. *Opt. Express* **19,** 15009 (2011).

13. Huang, F., Schwartz, S. L., Byars, J. M. & Lidke, K. A. Simultaneous multiple-emitter fitting for single molecule super-resolution imaging. *Biomed. Opt. Express* **2,** 1377 (2011).

14. Mukamel, E. A., Babcock, H. & Zhuang, X. Statistical deconvolution for superresolution fluorescence microscopy. *Biophys. J.* **102,** 2391–2400 (2012).

15. Gustafsson, N. *et al.* Fast live-cell conventional fluorophore nanoscopy with ImageJ through super-resolution radial fluctuations. *Nat. Commun.* **7,** (2016).

16. Wolter, S. *et al.* RapidSTORM: Accurate, fast open-source software for localization microscopy. *Nature Methods* **9,** 1040–1041 (2012).

17. Parthasarathy, R. Rapid, accurate particle tracking by calculation of radial symmetry centers. *Nat. Methods* **9,** 724–726 (2012).
